# The effectiveness of self-guided interventions in adults with depressive symptoms: a systematic review and meta-analysis

**DOI:** 10.1016/j.ebiom.2024.105208

**Published:** 2024-06-14

**Authors:** Lingyao Tong, Olga-Maria Panagiotopoulou, Pim Cuijpers, Eirini Karyotaki

**Affiliations:** a Department of Clinical, Neuro & Developmental Psychology, Amsterdam Public Health Research Institute, Vrije Universiteit Amsterdam, Amsterdam, the Netherlands; b WHO Collaborating Centre for Research and Dissemination of Psychological Interventions, Vrije Universiteit Amsterdam, Amsterdam, the Netherlands; c International Institute for Psychotherapy, Babeș-Bolyai University, Cluj-Napoca, Romania

**Keywords:** Self-guided interventions, Depression, Support levels, Treatment acceptability, Meta-analysis

## Abstract

**Background:**

Despite promising scalability and accessibility, evidence on the efficacy of self-guided interventions for adult depression is inconclusive. This study investigated their effectiveness and acceptability, considering diverse delivery formats and support levels.

**Methods:**

We systematically searched PubMed, PsycINFO, Embase, and Cochrane Library until 1st January 2024. Included were randomised controlled trials comparing self-guided interventions with a control condition for adult depression. Two independent researchers extracted data. Effect sizes were pooled using random-effects models, with post-intervention depressive severity compared with control conditions as the primary outcome. Study validity was evaluated using Cochrane Risk of Bias 2.0. This study was pre-registered with OSF (https://osf.io/rd43v).

**Findings:**

We identified 92 studies (111 interventions vs. control comparisons) with 16,706 participants (mean age: 18.78–74.41 years). Compared to controls, self-guided interventions were moderately effective at post-assessment (*g =* 0.53, 95% CI: 0.45–0.61; *I*^*2*^ = 79.17%) and six to twelve months post-randomisation follow-up (*g* = 0.32, 95% CI: 0.16–0.48; *I*^*2*^ = 79.19%). Trials with initial human screening (*g* = 0.59) and interventions delivered in computer programs (*g* = 1.04) had the significantly largest effect sizes. No differences in treatment effects were observed across support levels, therapy types, commercial availability, or the presence of online discussion forums. Self-guided interventions were less acceptable than control conditions (RR = 0.92, *p* < 0.001). Most studies showed a moderate to high risk of bias (n = 80).

**Interpretation:**

Existing trials on self-guided interventions are at high risk of bias, potentially overestimating treatment effects. Despite lower acceptability compared to controls, self-guided interventions are moderately effective in treating adult depression, regardless of support levels and online discussion features.

**Funding:**

None.

**Research in context**

**Evidence before this study**

Psychotherapy is a collaborative treatment approach based on the relationship between individuals and psychologists, typically tailored to personal goals. Self-guided interventions, in contrast to psychotherapy, are therapeutic approaches that individuals engage in independently, without therapeutic guidance. These innovative interventions provided flexibility, anonymity, and low cost, addressing many access-to-psychotherapy barriers. Currently, self-guided interventions vary in modality and support, including formats like self-help books, web-based programs, or mobile applications, with support levels ranging from none to weekly human encouragement calls. Through systematic searching in PubMed, PsycINFO, and Cochrane Library up to 1st January 2024, we found inconclusive evidence on the overall effectiveness of self-guided interventions for adult depression, ranging from large to moderate, moderate to small, and no effects. While some meta-analyses examined the effects of self-guided interventions in the context of different support levels, they specifically focused on written-formed cognitive-behavioural therapy (i.e., book and booklet) and internet-based interventions. A comprehensive examination of the various forms of self-guided interventions and their impact on treatment outcomes is still lacking in the current literature.

**Added value of this study**

This study presents comprehensive evidence of existing self-guided interventions in treating adults with depressive symptoms. It systematically investigates how various features of these interventions, such as delivery formats and support levels, correlate with treatment outcomes and adherence. Our findings suggest that different levels of support, including no support to weekly human calls, do not necessarily enhance treatment outcomes or adherence. However, we highlight the importance of initial human contact before the intervention, which significantly improves treatment effects and adherence.

**Implications of all the available evidence**

While self-guided interventions were moderately effective in treating adult depression, their acceptability was significantly lower than being in a control condition. Further research is needed to investigate factors contributing to the high dropout rates in these interventions, thus, improve intervention effectiveness. The evidence of the current study was at high risk of bias, which could impact the reliability and validity of the findings. There was also an under-representation of trials from low- and middle-income countries, even though access to mental health care is largely constrained, and self-administered interventions can be particularly promising for individuals in these regions. For practice, self-guided interventions can be a promising way to alleviate the need for licensed therapists, reduce financial burdens on individuals and governments, and mitigate the stigma associated with seeking mental health care. Clinicians can consider integrating these innovative approaches into regular face-to-face psychotherapy, given the prolonged waiting times.

## Introduction

Psychological interventions can effectively reduce adults’ depressive symptoms,^1^^,^^2^ whereas their accessibility is still restricted by a limited number of trained therapists, fear of stigmatisation, long waiting lists, and high treatment costs.^3^, ^4^, ^5^ Self-guided interventions, characterised as psychological interventions without therapeutic support, offer a promising way to increase access to evidence-based interventions.^6^^,^^7^ These innovative approaches often come in various formats, such as self-help books, mobile applications, or web-based programs; with multiple support levels, including no support, technical support, support on demand, and automated or human encouragement.^8^

Several meta-analyses have examined the efficacy of self-guided interventions for adult depression,^9^ demonstrating overall small to moderate effect sizes against control conditions at post-intervention.^6^, ^7^, ^8^, ^9^, ^10^, ^11^, ^12^ These effect sizes were found to be increased in studies examining internet-based interventions with therapeutic guidance.^11^, ^12^, ^13^, ^14^, ^15^, ^16^ Additionally, emerging research underscored the potential benefits of non-therapeutic support, such as human encouragement, in enhancing treatment adherence and efficacy.^12^^,^^16^ However, the existing literature on the dosage and response relationship in self-guided interventions remains scarce, particularly when considering the diverse contents and modalities, such as delivery formats, therapy types, and the pre- and post-intervention human contact.

Meta-analytic findings have indicated no differences in treatment efficacy between interventions offering minimal human support (e.g., technical support only) and those without any support.^11^^,^^12^^,^^17^ These studies specifically focused on internet-based psychological interventions,^11^^,^^12^ and written forms of cognitive-behavioural therapy (CBT).^17^ In addition, researchers compared system-generated automated encouragement and human encouragement in internet-based CBT, showing no significant differences in treatment outcomes.^8^ More recently, one study suggested increased effectiveness in online interventions involving human contact before and during the interventions.^18^ This study mixed therapist-guided and self-guided interventions and did not differentiate the degree of support provided during the interventions.

The current evidence on self-guided interventions predominantly concentrates on internet-based interventions, leaving out other forms such as computer programs, and video- and audio-based interventions. A gap of knowledge still exists regarding the effectiveness of self-guided interventions while considering their various features, such as different support levels and delivery formats. It also remains unknown whether and how these characteristics relate to treatment adherence. Therefore, the objective of this study was twofold: (1) to examine the overall effectiveness of self-guided interventions in treating adult depressive symptoms and (2) to investigate the potential impact of different self-guided intervention features on the estimates of treatment effect sizes. Moreover, considering the potential high dropout rates in self-guided interventions, we also examined the acceptability of self-guided interventions compared to the control conditions.

## Methods

### Search strategy and selection criteria

We included randomised controlled trials (RCTs) from an existing database on psychotherapy for depression (https://osf.io/825c6/). This meta-analytic database is updated every four months through systematic literature searches in PubMed, PsycINFO, Embase, and Cochrane Library (last update: 1st January 2024). The searches combined terms for depression and psychotherapies with filters for RCTs without language restrictions. The full search strings for PubMed are provided in the Supplement. Studies were also identified through searching in reference lists of previous meta-analyses, contacting other researchers, and from an individual patient data (IPD) database on internet-based interventions for depression and/or anxiety disorders (https://osf.io/p5emr/; last update: 6th February 2023). Grey literature was also consulted, including doctoral theses and protocols. Two independent researchers screened all records based on the titles and abstracts. Papers that could meet inclusion criteria according to one of them were further retrieved as full text. Any disagreements between the two researchers were resolved through discussion.

We included RCTs that compared any forms of self-guided interventions with a control condition (i.e., waitlist, usual care, or other inactive controls such as attention control). The self-guided interventions should focus on depressive symptoms and can provide necessary support to monitor the intervention process or improve participant adherence (e.g., technical support, automated or human encouragement); however, no support related to the therapeutic content was permitted. We only selected studies on adults (mean age ≥18 years) with elevated depressive symptoms based on validated clinical diagnostic interviews or self-reported scales (scoring above a cut-off). This inclusion criteria did not require depression to be the primary diagnosis, and participants with any type of physical or mental comorbidity were eligible. All major types of psychotherapies (e.g., CBT, problem-solving therapy, or third-wave therapy) delivered in any format (e.g., web-based, mobile-based, bibliotherapy) were eligible. We excluded single-session interventions as they were not comparable to the rest of the interventions and had high potential of increasing clinical heterogeneity.^19^ There were no restrictions on language.

### Data analysis

The validity of the included studies was assessed using the modified version of the Cochrane Risk of Bias (RoB) assessment tool, version 2,^20^ with detailed descriptions regarding the modifications provided in the Supplement. Five major sources of risk of bias were assessed, including (1) the randomisation process, (2) the deviations from the intended interventions, (3) the missing outcomes data, (4) the measurement of the outcome, and (5) the selection of reported results. In the end, studies were categorised as low risk, some concerns, and high risk. Two independent researchers conducted the evaluations. Any disagreements between them were solved through discussion.

We also coded participant characteristics (i.e., mean age, age group, sex indicated by the percentage of women, somatic or mental health comorbidities, diagnostic method, recruitment method, and target group), general characteristics of the interventions (i.e., type of intervention and the number of sessions planned), as well as the general characteristics of the studies (i.e., type of comparison, publication year, country where the study was conducted, self-reported or clinician-rated instruments, and dropout numbers from the study). In addition, we collected data specifically related to self-guided interventions, including delivery formats (i.e., web-based, computer-program, mobile apps, audio-based, video-based, bibliotherapy, and others), support levels (i.e., no support, technical support only, automated personalised or non-personalised encouragement, support on demand, and human encouragement), whether there was a face-to-face or telephone human contact at the start of the trial (i.e., yes or no), whether there was an online discussion forum (i.e., yes or no), and whether the intervention was commercially available by the time it was conducted (i.e., yes, no, or no information). The categorical criteria of different support levels were based on previous research,^8^ Detailed descriptions of each category relating to self-guided interventions are provided in the previous registration (https://osf.io/ut7n6/).

#### Statistics

The primary outcome was the severity of depression calculated by the standard mean differences between the intervention and control conditions at post-assessment. The long-term effect sizes after six months post-randomisation were also calculated when data were available. We used Hedges'g as the effect size considering many of the included studies have relatively small sample sizes.^21^ We also extracted data on participants' quality of life (QoL) and acceptability of the treatment when data was available. The QoL was examined only for psychological outcome at post-assessment and was assessed using Hedges'g.^21^ The acceptability of treatment was assessed by attrition rates at post-assessment. Participants failed to complete the post-assessments of depression scales for any reason were considered study dropouts. Relative risk (RR) with its 95% confidence interval (CI) was used as the treatment acceptability.

Meta-analyses were conducted using the metapsy Tools package (version 1.0.11) in Rstudio (version 4.2.2 for Mac). We pooled the effect sizes using several models to compare the robustness of estimated outcomes. In the main model, the effect sizes were calculated by first aggregating all effect sizes within one study (an intra-study correlation coefficient of *ρ* = 0.6 was assumed) and pooling the overall effects based on the arm level.^22^^,^^23^ Moreover, we conducted several sensitivity analyses to pool the effect sizes by (1) aggregating all effect sizes within one study and pooling the effects based on the study level, (2) only using the smallest or largest effect in each study, (3) only including studies with a low risk of bias, and (4) excluding studies outliers (defined as studies whose 95% CI of the effect size does not overlap with the 95% CI of the pooled effect size based on the main model). We also conducted a three-level correlated and hierarchical effects (CHE) model, which assumed that effect sizes were nested in studies and effects within studies are correlated (correlation coefficient *ρ* = 0.6).^22^^,^^23^ To test the robustness of the combined and three-level CHE models, sensitivity analyses were performed using different correlation coefficients (*ρ*-value).^22^ The standard *p* < 0.05 criteria was used to determine if differences between groups of studies were significant.

We calculated number-needed-to-be-treated (NNT) using Furukawa's formulae in which the control group's event rate was conservatively assumed to be 17%.^24^ Considering the high potential of publication bias caused by the bias of significant research outcomes,^25^ we conducted a funnel plot for a visual inspection of publication bias, with Egger's tests used to test whether the bias captured by the funnel plot was significant.^26^ Different publication bias methods were applied to assess and adjust for potential publication bias, including Duval and Tweedie's trim and fill procedure,^27^ Rücker's limit meta-analysis method,^28^ and the three-parameter selection model.^29^ The advantages and disadvantages of each publication bias method have been discussed thoroughly in a previous publication.^30^

We calculated the *I*^*2*^ statistic with 95% CI to quantify heterogeneity across the included studies, with an *I*^*2*^ of 25% low, 50% moderate, and 75% high heterogeneity.^31^ The prediction intervals (PI) were also calculated to indicate an expected range of effects in which future studies will fall.^32^ A random-effects model was used for all analyses, considering the potentially high heterogeneity expected in the study.

A series of subgroup analyses were conducted to examine the effects of the intervention based on the characteristics of the participants, the interventions, and the studies. Mixed-effects models were used in which studies within subgroups are pooled with the random-effects model, while differences between subgroups are tested with the fixed-effects model. Univariate meta-regression analyses were also undertaken to examine possible sources of heterogeneity and determine whether the effect sizes are associated with the year of publication, mean age, percentage of women, and the number of sessions planned for the intervention.

The protocol of the current meta-analysis was pre-registered at OSF (https://osf.io/rd43v) with R scripts available at https://osf.io/ut7n6/. The PRISMA checklist is available in the Supplement.

### Ethics

Institutional ethical approval was not required for this systematic review and meta-analysis.

### Role of funders

There was no funding source for this study.

## Results

### Study selection and characteristics

**Fig. 1:**
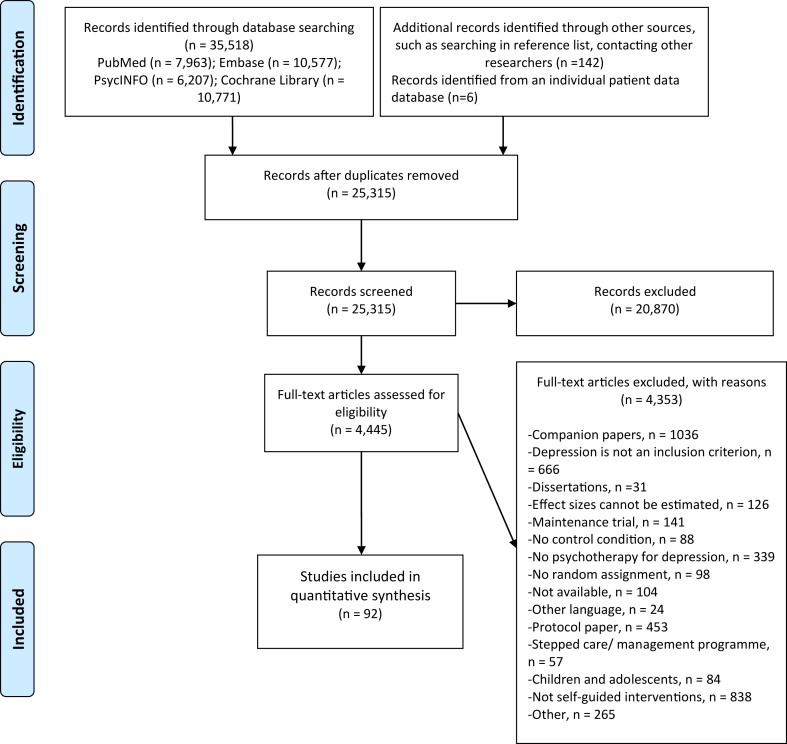
PRISMA flow diagram of study selection process.

After examining 35,518 records (25,309 after duplicates were removed), we retrieved 4439 full texts for further consideration. We identified 80 studies from the existing database on psychotherapy for depression (https://osf.io/825c6/) and 12 studies from the IPD meta-analytic database (https://osf.io/p5emr/). A total of 92 RCTs (111 comparisons between a self-guided intervention and a control condition) met the inclusion criteria with 16,706 participants (n = 8723 in intervention conditions and n = 7983 in control conditions) included in the data analyses.^d^ The PRISMA flowchart describing the selection process and exclusion reasons of the current study is presented in Fig. 1.

Selected characteristics of included studies with sex reports are presented in Supplement (Table S1). Most of the studies included were conducted in high-income countries (n = 82, 89.1%). Participants were mainly recruited from community settings (56 studies, 60.9%); they were also recruited from clinical settings (12 studies, 13.0%) or other settings, such as screening based on medical records in hospitals where participants were diagnosed by scoring above a cut-off score on a depression scale or meeting criteria for depression based on a clinical interview (24 studies, 26.1%). In contrast with participants recruited through community or clinical settings, those who were identified through screening did not seek mental health help actively.^33^ Most interventions were CBT-based (79 comparisons, 71.2%). Almost half of the control conditions were waitlists (53 comparisons, 47.7%); others were care-as-usual (n = 28, 25.2%) or other controls such as attention control (n = 30, 27.0%). Fifty-seven comparisons (51.4%) used web-based interventions; they were also mobile-based (n = 19, 17.1%), written-formatted (n = 20, 18.0%), computerised (n = 5, 4.5%), and other formats such as audio-, video-based, mail-based, or mixed formats (n = 10, 9.0%). Thirty-five interventions (31.5%) provided human encouragement such as weekly calls that aimed to improve adherence. Forty interventions (36.0%) did not provide any support. Only around a quarter of studies were commercial interventions by the time the study was conducted (n = 31, 27.9%). Twelve trials (13.0%) were rated as low risk of bias, thirty-eight trials (41.3%) were at a high risk of bias, and forty-two trials (45.7%) had an overall rating of some concerns (for detailed information, see Figure S3 for the risk of bias summary plot and Figure S4 for the traffic light plot).

### The overall effectiveness of self-guided interventions

We found a moderate treatment effect size of self-guided interventions on improving adults’ depressive symptoms (Table 1). In the main model (combined effect sizes based on all comparisons), the effect size at post-assessments was moderate (*g* = 0.53, 95% CI: 0.45–0.61). We found an NNT of 5.61, which indicates that we need to treat approximately six patients to improve one additional patient in practice compared to the control conditions. The heterogeneity of included studies was very high (*I*^2^ = 79.17%, 95% CI: 75.22–82.49), showing a high variation of the effect sizes between studies. The results of PI ranged from −0.16 to 1.22, indicating that further research might find a negative treatment outcome. There were 22 studies with 24 comparisons that did not overlap with the 95% CI of the combined effects, which were considered outliers (see the forest plot provided in the Supplement, Figure S1). A very high heterogeneity was found in these studies (*I*^2^ = 92.12%; 95% CI: 89.58–94.04). After excluding these outliers, the effect size slightly decreased to *g* = 0.52 (95% CI: 0.47–0.57) and the heterogeneity of included studies significantly decreased to *I*^2^ = 26.26% (95% CI: 3.22–43.82). We further explored the differences in study characteristics between the outliers and non-outlier groups, and we found no significant differences in study design, risk of bias, and participant and treatment characteristics (see Table S2 in Supplement). We found twelve studies with 13 comparisons were at low risk of bias. The effect size for these comparisons was small to moderate (*g* = 0.43, 95% CI: 0.24–0.63; NNT = 7.08).

**Table 1:** Overall effects of self-guided interventions for adult depression compared with control conditions.

	N	*g*	95% CI	*p*	*I*^*2*^	95% CI	PI	NNT
**Post-tests**								
Combined (all comparisons)	111	0.53	0.45–0.61	<0.001	79.17	75.22–82.49	−0.16 to 1.22	5.61
Combined (study level)	92	0.53	0.44–0.62	<0.001	80.86	76.94–84.12	−0.18 to 1.24	5.64
One ES/study (lowest only)	92	0.46	0.37–0.55	<0.001	76.29	71.1–80.55	−0.21 to 1.13	6.61
One ES/study (highest only)	92	0.63	0.52–0.74	<0.001	85.64	82.94–87.9	−0.31 to 1.57	4.62
Outliers removed	87	0.52	0.47–0.57	<0.001	26.26	3.22–43.82	0.27 to 0.76	5.81
Only low risk of bias	13	0.43	0.24–0.63	<0.001	75.73	58.39–85.84	−0.21 to 1.08	7.08
Three-Level Model (CHE)	156	0.56	0.47–0.65	<0.001	84.30	–	−0.24 to 1.36	5.29
**Publication bias correction at post-tests**								
Trim-and-fill method (41 studies added)	152	0.29	0.19–0.39	<0.001	85.9	83.89–87.65	−0.79 to 1.37	11
Limit meta-analysis	111	0.33	0.21–0.45	<0.001	85.98	–	−0.36 to 1.03	9.52
Selection model	111	0.52	0.44–0.61	<0.001	83.35	76.34–88.87	−0.18 to 1.23	5.73
**Follow-up tests (6 to 12 months post randomization)**								
Combined (all comparisons)	31	0.32	0.16–0.48	<0.001	79.19	71.03–85.05	−0.45 to 1.08	9.98
Combined (study level)	25	0.33	0.14–0.51	0.001	82.12	74.52–87.45	−0.52 to 1.17	9.69
One ES/study (lowest only)	25	0.26	0.09–0.44	0.005	74.80	62.82–82.92	−0.47 to 1	12.19
One ES/study (highest only)	25	0.31	0.15–0.46	<0.001	71.47	57.36–80.91	−0.22 to 0.83	10.43
Outliers removed	29	0.27	0.17–0.36	<0.001	48.96	21.46–66.83	−0.05 to 0.58	12.11
Only low risk of bias	5	0.35	0.01–0.69	0.045	69.34	21.44–88.04	−0.45–1.15	8.9
Three-Level Model (CHE)	49	0.29	0.14–0.44	<0.001	81.70	–	−0.32 to 0.91	10.93
**Publication bias correction at follow-up tests**								
Trim-and-fill method (6 studies added)	37	0.17	−0.02–0.36	0.081	85.71	81.24–89.11	−0.91 to 1.25	19.68
Limit meta-analysis	31	0.22	−0.02–0.47	0.077	89.05	–	−0.57 to 1.02	14.64
Selection model	31	0.24	−0.01–0.49	0.060	90.34	79.2–96.58	−0.63 to 1.11	13.62

N, numbers of comparisons; ES, effect size; n, number of comparisons; CI, confidence interval; PI, prediction interval; NNT, numbers-needed-to-be-treated.

We found considerable publication bias through visual inspection of the funnel plot (Supplement, Figure S2), with Egger's test also pointing out a significant asymmetry (intercept: 1.96; 95% CI: 1.30–2.62, *p* < 0.001). When correcting for publication bias (Table 1), distinct effect sizes were found across three test methods, as the selection model showed comparable effect sizes (*g* = 0.52, 95% CI: 0.44–0.61; NNT = 5.73) with the main model; however, limit meta-analysis (*g* = 0.33, 95% CI: 0.21–0.45; NNT = 9.52) and Duval & Tweedie's trim and fill method (41 studies added, *g* = 0.29, 95% CI: 0.19–0.39; NNT = 11) showed a largely decreased but still significant effect sizes compared to the main model. We found very comparable results while using difference correlation coefficients in the combined and three-level CHE models, with results provided in the Supplement (Table S3).

We identified 25 studies (31 comparisons) reporting long-term treatment effects (six to 12 months post-randomisation; Table 1). Overall, the effect size of self-guided interventions at follow-up was small to moderate (*g* = 0.32, 95% CI: 0.16–0.48; NNT = 9.98). The heterogeneity of included studies remained very high (*I*^*2*^ = 79.19%; 95% CI: 71.03–85.05).

### Subgroup analyses

For the specific characteristics of self-guided interventions, subgroup analyses showed a significantly larger effect size in trials that had an initial human screening (*g* = 0.59, 95% CI: 0.48–0.71) compared to no human screening (*g* = 0.41, 95% CI: 0.32–0.49; *p* = 0.007; Table 2). Interventions delivered by a computer program showed the largest effect size (*g* = 1.04, 95% CI: 0.32–1.76) compared to mobile-based interventions (*g* = 0.73, 95% CI: 0.43–1.04), written-format interventions (*g* = 0.65, 95% CI: 0.44–0.86), web-based intervention (*g* = 0.44, 95% CI: 0.36–0.53), or other format such as video- or audio-based interventions (*g* = 0.35, 95% CI: 0.07–0.63; *p* = 0.015). There were no statistically significant subgroup differences in support levels, commercial availability, or the presence of an online discussion forum.

**Table 2:** Subgroup analyses of self-guided intervention for adult depression compared with control conditions at post-assessments.

	N	*g*	95% CI	*I*^*2*^	95% CI	NNT	*P*
Risk of bias							
High risk	49	0.63	0.51–0.75	68.9	58.4–76.8	4.63	0.063
Some concerns	49	0.49	0.36–0.62	82.7	77.9–86.5	6.15	
Low risk	13	0.43	0.24–0.63	75.73	58.39–85.8	7.08	
Control group							
CAU	28	0.35	0.23–0.48	71.5	58.4–80.5	8.97	**<0.001**
Waitlist	53	0.71	0.58–0.84	72.8	64.3–79.3	4.04	
Other	30	0.42	0.28–0.55	79.7	71.7–85.5	7.32	
Age group							
Adults	89	0.53	0.43–0.62	81.1	77.2–84.3	5.63	0.449
Young adults	13	0.62	0.45–0.80	53.1	12.0–75.0	4.71	
Older adults	9	0.47	0.20–0.73	58.9	14.2–80.3	6.45	
Recruitment							
Clinical	14	0.35	0.15–0.54	76.2	60.1–85.8	8.97	**0.017**
Community	71	0.61	0.50–0.72	78.7	73.5–82.9	4.80	
Other^a^	26	0.42	0.29–0.56	71.0	57.0–80.5	7.32	
Target group							
Unselected adults	64	0.51	0.41–0.61	80.0	74.9–84.0	5.88	0.566
Student	11	0.65	0.39–0.91	72.7	49.9–85.1	4.46	
PPD Women	8	0.78	0.04–1.52	90.3	83.3–94.4	3.63	
General medical	9	0.44	0.19–0.70	81.7	66.5–90.1	6.95	
Other^b^	19	0.46	0.29–0.62	53.2	21.2–72.2	6.61	
Diagnosis							
Cut-off	79	0.51	0.41–0.60	74.4	68.1–79.4	5.88	**0.013**
Depressive disorder^c^	26	0.67	0.48–0.85	84.6	78.6–89.0	4.31	
Subclinical	6	0.26	0.00–0.53	76.6	47.7–89.5	12.44	
Mental health comorbidity							
Yes	3	0.26	0.18–0.70	2.7	0.00–89.9	12.44	**0.011**
No	108	0.54	0.46–0.62	79.6	75.7–82.9	5.51	
Somatic comorbidity							
Yes	9	0.45	0.19–0.71	81.7	66.4–90.0	6.77	0.450
No	102	0.54	0.45–0.63	79.0	74.8–82.5	5.51	
Rating instrument							
Self-reported	92	0.50	0.41–0.59	79.4	75.1–83.0	6.01	0.082
Clinician-rated	19	0.69	0.48–0.91	63.3	40.0–77.6	4.17	
Type of therapy							
CBT	79	0.55	0.46–0.64	80.2	75.7–83.8	5.40	0.492
Other	32	0.49	0.33–0.65	76.9	67.7–83.5	6.15	
Support level							
No support	40	0.47	0.32–0.62	75.2	66.4–81.7	6.45	0.419
Technical support	12	0.50	0.23–0.77	87.8	80.5–92.3	6.01	
Automated encouragement	16	0.56	0.29–0.84	88.7	83.3–92.4	5.29	
Support on demand	8	0.47	0.36–0.58	0.00	0.00–67.6	6.45	
Human encouragement	35	0.62	0.49–0.75	71.3	59.7–79.5	4.71	
Delivery format							
Web-based	57	0.44	0.36–0.53	79.6	74–84	6.95	**0.015**
Mobile-based	19	0.73	0.43–1.04	79.2	68.2–86.4	3.91	
Computer program	5	1.04	0.32–1.76	74.9	38.2–89.8	2.64	
Bibliotherapy	20	0.65	0.44–0.86	71.3	54.9–81.7	4.46	
Other^d^	10	0.35	0.07–0.63	70.9	44.5–84.8	8.97	
Online forum							
Yes	11	0.43	0.29–0.57	16.5	0.00–56.9	7.13	0.138
No	100	0.54	0.45–0.63	80.8	77.1–84.0	5.51	
Initial human screening							
Yes	77	0.59	0.48–0.71	81.7	77.6–85.0	4.9	**0.007**
No	34	0.41	0.32–0.49	65.9	51.1–76.2	7.52	
Commercial availability							
Yes	31	0.62	0.48–0.77	80.3	72.6–85.7	4.71	0.144
No/No information	80	0.50	0.4–0.59	78.6	73.7–82.6	6.01	

N, numbers of comparisons; CI, confidence interval; NNT, numbers-needed-to-be-treated; CAU, care as usual; CBT, cognitive behavior therapy; PPD, postpartum depression.

Bold numbers indicate statistical significant restuls (*p* < 0.05).

a Other recruitment methods = methods such as screening, recruitment from known patients in general medical settings, or recruitment method that was not described in the paper.

b Other target groups = older adults above 50 years old, or any other specific target group.

c Depressive disorder = clinician-rated major depression disorders, mood disorders, and chronic depression.

d Other delivery formats = audio-based, video-based, mail-based, or mixed modes of delivery.

In addition, trials recruited samples from community settings had larger effect sizes (*g* = 0.61, 95% CI: 0.50–0.72) than from clinical settings (*g* = 0.35, 95% CI: 0.15–0.54) or other settings such as screening (*g* = 0.42, 95% CI: 0.29–0.56; *p* = 0.017; Table 2 subgroup analyses). Studies using a waitlist control group showed a larger effect size (*g* = 0.71, 95% CI: 0.58–0.84) than usual care (*g* = 0.35, 95% CI: 0.23–0.48) or other control conditions (*g* = 0.42, 95% CI: 0.28–0.55; *p* < 0.001). Individuals with clinician diagnoses also presented a larger effect size (*g* = 0.67, 95% CI: 0.48–0.85) than those who used a cut-off score (*g* = 0.51, 95% CI: 0.41–0.60) or subclinical depression (*g* = 0.26, 95% CI: 0.00–0.53; *p* = 0.013). Participants with any types of mental health comorbidities showed a significantly lower level of treatment efficacy (*g* = 0.26, 95% CI: 0.18–0.70), comparing to the group without co-occurring metal health disorders (*g* = 0.54, 95% CI: 0.46–0.62). These findings should be considered with caution, however, because only three studies focused on comorbid conditions. No significant subgroup differences were found in the trials’ risk of bias, age group, target group, rating instrument, comorbidities with somatic disorders, and type of intervention.

In meta-regression analyses, results showed a significant and negative association between mean age and post-intervention depression (estimate: −0.01, *p* < 0.05; mean age range from 18.78 to 74.41 years). No other significant findings were found, including year of publication, number of intervention sessions planned, or percentage of women.

Considering the potential high dropout rates of self-guided interventions, we measured participant attritions of studies at post-assessment as treatment acceptability. The average dropout rates were 23.9% for self-guided interventions and 17.9% for control conditions. As can be seen from Table 3, self-guided interventions were overall less acceptable than being in a control condition (RR = 0.92, 95% CI: 0.87–0.96; *p* < 0.001). Furthermore, subgroup analyses revealed significantly less acceptability in web-based interventions (RR = 0.85; *p* = 0.014) and studies without initial human screening (RR = 0.77; *p* = 0.007). There were no subgroup differences in support levels or online discussion features. Lastly, sixteen studies (16 comparisons) assessed psychological QoL at post-assessment, showing an overall moderate effect size (*g* = 0.31, 95% CI: 0.17–0.45; NNT = 10.24).

**Table 3:** Treatment acceptability of self-guided interventions at post-assessments.

	N	RR	95% CI	*I*^*2*^	95% CI	*p*^*1*^	*p*^*2*^
**Overall**	111	0.92	0.87–0.96	77.0	72.5–80.7	**<0.001**	
**Support levels**							0.715
No support	40	0.95	0.91–0.99	53.9	34.0–67.8	0.015	
Technical support	12	0.78	0.47–1.27	90.4	85.1–93.8	0.028	
Automated encouragement	16	0.84	0.66–1.07	88.5	82.9–92.2	0.150	
Support on demand	8	0.93	0.81–1.06	79.3	59.6–89.4	0.213	
Human encouragement	35	0.95	0.92–0.99	52.7	30.4–67.8	0.013	
**Delivery formats**							**0.014**
Web-based	57	0.85	0.77–0.94	83.1	78.8–86.6	**0.002**	
Mobile-based	19	0.98	0.93–1.03	45.0	5.8–67.9	0.391	
Computer program	5	0.97	0.78–1.19	27.2	0.0–71.3	0.732	
Bibliotherapy	20	0.97	0.93–1.01	29.0	0.0–58.8	0.207	
Other	10	1.00	0.99–1.01	0.0	0.0–62.4	0.928	
**Initial human screening**							**0.007**
Yes	77	0.97	0.95–1.00	57.4	45.1–66.9	**0.029**	
No	34	0.77	0.64–0.92	86.8	82.6–90.0	**0.005**	
**Online forum**							0.073
Yes	11	0.70	0.49–1.01	89.4	83.0–93.3	0.054	
No	100	0.94	0.91–0.98	71.6	65.4–76.7	**0.002**	

N, numbers of comparisons; RR, risk ratio; CI, confidence interval.

Bold numbers indicate statistically significant results (*p* < 0.05).

***p***^***1***^ = within-group *p*-value testing the differences between the intervention and control condition, ***p***^***2***^ = between-group *p*-value testing the differences across subgroups (random effects model).

## Discussion

This meta-analysis assessed the effectiveness of self-guided interventions for adults with depressive symptoms, considering various trial features, participant characteristics, and intervention attributes. The interventions examined were predominantly based on CBT, delivered via the Internet, and were non-profit programs without support or online discussion forums. The effectiveness of these self-guided approaches for adult depression at post-assessment was moderate, aligning with previous meta-analytic findings.^7^, ^8^, ^9^, ^10^, ^11^ Although these effects slightly decreased to a small-to-moderate level during follow-up assessments, they remained statistically significant up to 12 months.^6^ The follow-up effect sizes are comparable to those interventions with therapeutic guidance,^14^ suggesting their potential for maintaining long-term treatment efficacy.

A significantly larger treatment effect was found in trials with initial human screening.^16^^,^^18^ These findings are in line with the Supportive Accountability theory, indicating that human contact can promote participants' feelings of accountability and, thus, improve treatment adherence and outcomes.^34^ In addition, the impact of human contact in self-guided interventions appeared more significant during enrolment than post-intervention, as there were no differences in treatment effects between self-reported questionnaires and clinician-assessed interviews conducted after the intervention. Notably, the significant larger efficacy found in the computer program demands careful consideration, given that only five trials were included in the subgroup analyses. Half of these trials were conducted in an onsite setting with the presence of research staff, which could improve participants’ engagement and consequent treatment efficacy.^34^

Furthermore, our results suggested comparable treatment outcomes across various levels of support, ranging from no support to weekly human calls. These findings are consistent with previous research,^8^^,^^11^^,^^17^ whereas the reason remains uncertain. Regarding treatment dropout, self-guided interventions were less acceptable than being in a control condition.^12^^,^^13^ The study dropout rates are significantly higher in web-based interventions and trials without initial human screening. Treatment acceptability did not improve when participants were provided with enhanced support, such as weekly human encouragement calls or an online discussion forum. These findings suggest the promising scalability of self-guided interventions, as they maintain similar efficacy and acceptability while minimising or even eliminating certain types of support.

Lastly, in line with previous research, a larger treatment effect estimate was found in studies that compared to waitlist controls,^35^ recruited participants from community settings,^1^ diagnosed by clinicians,^36^ and individuals without any mental health comorbidities.^37^ No statistical differences were found in treatment effect sizes regarding the presence of an online discussion forum, whether the intervention was a commercial program, or types of psychotherapy (CBT vs. others). No session-and-effects relationships (N sessions ranged from 3 to 20) were found in the current analyses, indicating the possibility of delivering self-guided interventions at minimal intensity.

### Caveats and limitations

Several limitations of the current study are notable. First, only 12 studies (13.0%) included in the current analyses were at low risk of bias, which has the potential to overestimate the current effect sizes.^34^ We did not assess the inter-rater reliability for both the record screening process and the risk of bias assessment, which could potentially impact the reliability of the present findings. Second, there was a lack of long-term assessment on symptoms of depression outcomes, especially for the effects after one-year. Third, the heterogeneity of included studies was very high (*I*^*2*^ = 79.17), showing a high variability across the trials. Further, we observed considerable publication bias, with effect sizes changed from g = 0.53 to 0.29 after 41 studies added in Duval & Tweedie's trim and fill method. Last, nearly 90% of the included studies are from high-income countries, generalising the findings of this study to low- and middle-income countries need caution.

In conclusion, self-guided interventions are promising in scaling up and de-stigmatisation in a real-world setting. These approaches can be particularly useful for individuals who have limited access to regular therapy or fear of stigma. However, It is still vital to keep in mind that these interventions may not be suitable for all individuals or mental health concerns. Previous evidence indicated a higher treatment effect among individuals with mild-to-moderate depressive symptoms compared to those with severe symptoms,^14^ suggesting the potential need for specialised treatment and supervision from a trained therapist for those with severe symptoms. Individuals or therapists implementing self-guided interventions should carefully evaluate the benefits and potential risks, seeking or offering guidance as needed.

## Contributors

EK and PC conceived the study. EK, PC, OP, and LT designed the project and protocols. EK and PC did the literature search and screened the records. LT and OP extracted the data and assessed the study validity. LT analysed the data and wrote the manuscript. PC, EK, and OP reviewed and edited the manuscript. EK and PC supervised this project. All authors read and approved the final version of the manuscript.

## Data sharing statement

Study data will be publicly available on the Metapsy website (https://www.metapsy.org/) right upon publication.

## Declaration of interests

We declare no competing interests.

## Footnotes

d The detailed number of participants assigned to each condition is provided in Supplement, Table S1.
