# Supplementary material for: The effectiveness of self-guided interventions in adults with depressive symptoms: a systematic review and meta-analysis
Source: eBioMedicine. 2024 Jun 14;105:105208. doi: 10.1016/j.ebiom.2024.105208 (PMC11226978; doi:10.1016/j.ebiom.2024.105208)
Supplement: Supplement.docx [file mmc1.docx]

**Supplemental Online Content**

The effectiveness of self-guided interventions in adults with depressive symptoms: a systematic review and meta-analysis

Lingyao Tong, Olga-Maria Panagiotopoulou, Pim Cuijpers, and Eirini Karyotaki

Table of Contents

[A. Search string for PubMed 2](#_Toc167023280)

[1. Search string for PubMed in psychotherapy for depression database: 2](#_Toc167023281)

[2. Search string for PubMed in E-health (IPDMA) database: 4](#_Toc167023282)

[B. Modified manual of Cochrane risk of bias Tool, version 2. 7](#_Toc167023283)

[C. Supplemented Tables & Figures 9](#_Toc167023284)

[Table S1. selected characteristics of included studies. 9](#_Toc167023285)

[Table S2. Significance test of difference in study characteristics between outliers (n studies=22) and non-outliers (n studies=70). 25](#_Toc167023286)

[Table S3. Sensitivity analyses testing the impact of different correlation coefficients on the pooled effect sizes. 26](#_Toc167023287)

[Figure S1. Forest plot. 27](#_Toc167023288)

[Figure S2. Funnel plot. 28](#_Toc167023289)

[Figure S3. Risk of Bias Summary Plot. 29](#_Toc167023290)

[Figure S4. Risk of Bias traffic light plot. 30](#_Toc167023291)

[d. PRISMA CHEKLIST 31](#_Toc167023292)

[E. References of included studies 34](#_Toc167023293)

# A. Search string for PubMed

## 1. Search string for PubMed in psychotherapy for depression database:

(Psychotherapy MH OR psychotherap*All Fields OR cbtAll Fields OR "behavior therapies"All Fields OR "behavior therapy"All Fields OR "behavior therapeutic"All Fields OR "behavior therapeutical"All Fields OR "behavior therapeutics"All Fields OR "behavior therapeutist"all Fields OR "behavior therapeutists"All Fields OR "behavior treatment"All Fields OR "behavior treatments"All Fields OR "behaviors therapies"All Fields OR "behaviors therapy"All Fields OR "behaviors therapeutics"All Fields OR "behaviors therapeutic"All Fields OR "behaviors therapeutical"All Fields OR "behaviors therapeutist"All Fields OR "behaviors therapeutists"All Fields OR "behaviors treatment"All Fields OR "behaviors treatments"All Fields OR "behavioral therapies"All Fields OR "behavioral therapy"All Fields OR "behavioral therapeutics"All Fields OR "behavioral therapeutic"All Fields OR "behavioral therapeutical"All Fields OR "behavioral therapeutist"All Fields OR "behavioral therapeutists"All Fields OR "behavioral treatment"All Fields OR "behavioral treatments"All Fields OR "behaviour therapies"All Fields OR "behaviour therapy"All Fields OR "behaviour therapeutic"All Fields OR "behaviour therapeutical"All Fields OR "behaviour therapeutics"All Fields OR "behaviour therapeutist"all Fields OR "behaviour therapeutists"All Fields OR "behaviour treatment"All Fields OR "behaviour treatments"All Fields OR "behaviours therapies"All Fields OR "behaviours therapy"All Fields OR "behaviours therapeutics"All Fields OR "behaviours therapeutic"All Fields OR "behaviours therapeutical"All Fields OR "behaviours therapeutist"All Fields OR "behaviours therapeutists"All Fields OR "behaviours treatment"All Fields OR "behaviours treatments"All Fields OR "behavioural therapies"All Fields OR "behavioural therapy"All Fields OR "behavioural therapeutics"All Fields OR "behavioural therapeutic"All Fields OR "behavioural therapeutical"All Fields OR "behavioural therapeutist"All Fields OR "behavioural therapeutists"All Fields OR "behavioural treatment"All Fields OR "behavioural treatments"All Fields OR "cognition therapies"All Fields OR "cognition therapie"All Fields OR "cognition therapy"All Fields OR "cognition therapeutical"All Fields OR "cognition therapeutic"All Fields OR "cognition therapeutics"All Fields OR "cognition therapeutist"All Fields OR "cognition therapeutists"All Fields OR "cognition treatment"All Fields OR "cognition treatments"All Fields OR psychodynamicAll Fields OR PsychoanalysisMH OR psychoanalysisAll Fields OR psychoanalytic*All Fields OR counsellingAll Fields OR counselingAll Fields OR CounselingMH OR "problem-solving"All Fields OR mindfulnessAll Fields OR (acceptanceAll Fields AND commitmentAll Fields ) OR "assertiveness training"All Fields OR "behavior activation"All Fields OR "behaviors activation"All Fields OR "behavioral activation"All Fields OR "cognitive therapies"All Fields OR "cognitive therapy"All Fields OR "cognitive therapeutic"All Fields OR "cognitive therapeutics"All Fields OR "cognitive therapeutical"All Fields OR "cognitive therapeutist"All Fields OR "cognitive therapeutists"All Fields OR "cognitive treatment"All Fields OR "cognitive treatments"All Fields OR "cognitive restructuring"All Fields OR (("compassion-focused"All Fields OR "compassion-focussed"All Fields) AND (therapySH OR therapiesAll Fields OR therapyAll Fields OR therape*All Fields OR therapis*All FieldsOR Therapeutics OR treatment*All Fields)) OR ((therapySH OR therapiesAll Fields OR therapy All Fields OR therape*All Fields OR therapis*All Fields OR TherapeuticsMH OR treatment*All Fields) AND constructivist*All Fields) OR "metacognitive therapies"All Fields OR "metacognitive therapy"All Fields OR "metacognitive therapeutic"All Fields OR "metacognitive therapeutics"All Fields OR "metacognitive therapeutical"All Fields OR "metacognitive therapeutist"All Fields OR "metacognitive therapeutists"All Fields OR "metacognitive treatment"All Fields OR "metacognitive treatments"All Fields OR "meta-cognitive therapies"All Fields OR "meta-cognitive therapy"All Fields OR "meta-cognitive therapeutic"All Fields OR "meta-cognitive therapeutics"All Fields OR "meta-cognitive therapeutical"All Fields OR "meta-cognitive therapeutist"All Fields OR "meta-cognitive therapeutists"All Fields OR "meta-cognitive treatment"All Fields OR "meta-cognitive treatments"All Fields OR "solution-focused therapies"All Fields OR "solution-focused therapy"All Fields OR "solutionfocused therapeutic"All Fields OR "solution-focused therapeutics"All Fields OR "solution-focused therapeutical"All Fields OR "solution focused therapies"All Fields OR "solution focused therapy"All Fields OR "solution focused therapeutic"All Fields OR "solution focused therapeutics"All Fields OR "solution focused therapeutical"All FieldsOR "solution-focussed therapies"All Fields OR "solution-focussed therapy"All Fields OR "solution-focussed therapeutic"All Fields OR "solution-focussed therapeutics"All Fields OR "solution-focussed therapeutical"All FieldsOR "solution focussed therapies"All Fields OR "solution focussed therapy"All Fields OR "solution focussed therapeutic"All Fields OR "solution focussed therapeutics"All Fields OR "solution focussed therapeutical"All Fields OR "self-control therapies"All Fields OR "self-control therapy"All Fields OR "self-control therapeutics"All Fields OR "self-control therapeutical"All Fields OR "self-control therapeutic"All Fields OR "self-control training"All Fields OR "self-control trainings"All Fields OR "self control therapies"All Fields OR "self control therapy"All Fields OR "self control therapeutics"All Fields OR "self control therapeutical"All Fields OR "self control © 2020 American Medical Association. All rights reserved. therapeutic"All Fields OR "self control training"All Fields OR "self control trainings"All Fields AND (Depressive DisorderMH OR DepressionMHOR dysthymi*All Fields OR "affective disorder"All FieldsOR "affective disorders"All Fields OR "mood disorder"All Fields OR "mood disorders"All Fields OR depression*All Fields OR depressive*All Fields OR "dysthymic disorder"MeSH Terms) AND ((randomized controlled trial pt OR controlled clinical trial pt OR randomized tiab OR randomly tiab NOT (animalsmh NOT (animalsmh AND humans mh))

## 2. Search string for PubMed in E-health (IPDMA) database:

# Depression

"Depression"Mesh OR "Depressive Disorder"Mesh OR “depress*”tiab OR “dysthymi*”tiab OR “mood disorder*”tiab OR “affective disorder*”tiab OR “dysphoric disorder*”tiab

#Anxiety

“Anxiety disorders” Mesh OR "Anxiety"Mesh OR "Fear"Mesh OR "shyness"MeSH Terms OR shynesstiab OR shytiab OR “anxiet*”tiab OR “agoraphobi*”tiab OR “panic”tiab OR “social phobi*”tiab OR “phobi*”tiab OR “obsessive-compulsive”tiab OR “neurotic disorder*”tiab OR “hoarding*”tiab OR “OCD”tiab OR “neurotic anxiet*”tiab OR “anxious*”tiab OR “emotional disorder*”tiab OR feartiab OR worrytiab OR worryingtiab OR worriestiab OR GADtiab OR Arachnophobiatiab OR Ophidiophobiatiab OR Acrophobiatiab OR Cynophobiatiab OR Claustrophobiatiab OR Mysophobiatiab OR Aerophobiatiab OR Trypophobiatiab OR Carcinophobiatiab OR Thanatophobiatiab OR Glossophobiatiab OR Monophobiatiab OR Ornithophobiatiab OR Alektorophobiatiab OR Trypanophobiatiab OR Anthropophobiatiab OR Aquaphobiatiab OR Autophobiatiab OR Hemophobiatiab OR Xenophobiatiab OR Ailurophobiatiab OR Nyctophobiatiab OR Phobophobiatiab OR Philophobiatiab OR Triskaidekaphobiatiab OR Emetophobiatiab OR Entomophobiatiab OR Zoophobiatiab OR Scelerophobiatiab OR Cibophobiatiab OR Tokophobiatiab OR Pseudodysphagiatiab OR Gerascophobiatiab OR Technophobiatiab OR Ergophobiatiab OR Coulrophobia tiab OR Photophobiatiab OR Numerophobiatiab OR Taphophobiatiab

# Psychological interventions

“Psychotherapy”Mesh OR “Counseling”Mesh OR psychotherap*Tiab OR cbtTiab OR counsellingTiab OR counselingTiab OR “Eye Movement Desensitization Reprocessing”tiab OR “Eye Movement Desensitization and Reprocessing”tiab OR “Eye Movement Desensitisation Reprocessing”tiab OR “Eye Movement Desensitisation and Reprocessing”tiab OR EMDRtiab OR “Bibliotherap*”tiab OR mindfulnessTiab OR “Autogenic Training”tiab OR Logotherap*tiab OR "cognitive restructuring"Tiab OR "self-control training*"Tiab OR "assertiveness training"Tiab OR ((“therapy”SubHeading OR therap*Tiab OR “Therapeutics”Mesh OR treatment*Tiab OR intervention*tiab) AND (“brief psychodynamic”Tiab OR “short psychodynamic”tiab OR "problem-solving"Tiab OR "compassion-focused"Tiab OR "compassion-focussed"Tiab OR “compassion-based”tiab OR constructivist*Tiab OR metacognitivetiab OR “meta-cognitive”Tiab OR "solution-focused"Tiab OR "solution-focussed"Tiab OR "self-control”Tiab OR psychosocialtiab OR “peer support“tiab OR “task-shifted”tiab OR Relaxationtiab OR “dialectical behavior”tiab OR “emotion-focused”tiab OR narrativetiab OR “person-centred”tiab OR “person-centered”tiab OR “Narrative”tiab OR “meaning-centered”tiab OR “humanistic”tiab OR “client-centered”tiab OR “meaning-centred”tiab OR “client-centred”tiab OR “Rogerian”tiab OR “Nondirective”tiab OR “Non-directive”tiab OR “Supportive”tiab OR “Life review”tiab OR "acceptance and commitment"Tiab OR (“schema”tiab AND brieftiab) OR (“gestalt”tiab AND brieftiab))) OR "behavior therap*"Tiab OR "behaviors therap*"Tiab OR "behavioral therap*"Tiab OR "behaviour therap*"Tiab OR "behaviours therap*"Tiab OR "behavioural therap*"Tiab OR "cognition therap*"Tiab OR “cognitive therap*”tiab OR "behavior treatment*"Tiab OR "behaviors treatment*"Tiab OR "behavioral treatment*"Tiab OR "behaviour treatment*"Tiab OR "behaviours treatment*"Tiab OR "behavioural treatment*"Tiab OR "cognition treatment*"Tiab OR “cognitive treatment*”tiab OR "behavior intervention*"Tiab OR "behaviors intervention*"Tiab OR "behavioral intervention*"Tiab OR "behaviour intervention*"Tiab OR "behaviours intervention*"Tiab OR "behavioural intervention*"Tiab OR "cognition intervention*"Tiab OR “cognitive intervention*”tiab OR "behavior activation*"Tiab OR "behaviors activation*"Tiab OR "behavioral activation*"Tiab OR "behaviour activation*"Tiab OR "behaviours activation*"Tiab OR "behavioural activation*"Tiab OR exposuretiab

# Internet-based

"Telemedicine"Mesh OR "Mobile Applications"Mesh OR "Social Media"Mesh OR "Therapy, Computer-Assisted"Mesh:NoExp OR "Drug Therapy, Computer-Assisted"Mesh:NoExp OR "Telecommunications"Mesh:NoExp OR "Electronic Mail"Mesh OR "Videoconferencing"Mesh OR "Cell Phone"Mesh OR "Distance Counseling"Mesh OR “Wearable Electronic Devices”Mesh OR telehealthtiab OR “tele-health”tiab OR telepsychologytiab OR “tele-psychology”tiab OR telepsychiatrytiab OR “tele-psychiatry”tiab OR “tele-therap*”tiab OR teletherap*tiab OR “tele-medicine”tiab OR telemedicinetiab OR telecaretiab OR “tele-care”tiab OR telecommunicat*tiab OR “tele-communicat*”tiab OR teleconference*tiab OR “tele-conferenc*”tiab OR videoconferenc*tiab OR “video-conferenc*”tiab OR computer*tiab OR electronic*tiab OR digital*tiab OR ehealthtiab OR “e-health”tiab OR “e-treat*”tiab OR “e-therap*”tiab OR mhealthtiab OR “m-health”tiab OR “internet-based*”tiab OR “internet treat*”tiab OR “internet intervention*”tiab OR “internet counsel*”tiab OR “distance counsel*”tiab OR “web-based*”tiab OR cybercounsel*tiab OR “cyber-counsel*”tiab OR “online treat*”tiab OR “online therap*”tiab OR “online intervention*”tiab OR “online prevention*”tiab OR “online counsel*”tiab OR “text-messag*”tiab OR textmessag*tiab OR SMStiab OR texting*tiab OR “short message service*”tiab OR mobile*tiab OR smartphone*tiab OR “cell-phone*”tiab OR cellphone*tiab OR “cellular phone*”tiab OR blended*tiab OR “software app*”tiab OR “handheld device*”tiab OR “hand held device*”tiab OR iPad*tiab OR iPhone*tiab OR email*tiab OR “e-mail*”tiab OR sensor*tiab OR wearable*tiab OR “social media*”tiab OR “social network*”tiab OR “e-counsel*”tiab OR ecounsel*tiab OR palmtop*tiab OR telephone*tiab OR WhatsApptiab OR Twittertiab OR Facebooktiab OR Instagramtiab OR forumtiab OR chat*tiab OR “virtual reality*”tiab OR avatar*tiab OR “Conversational agent*”tiab OR “virtual coach”tiab OR “virtual agent*”tiab OR “embodied agent*”tiab OR “relational agent*”tiab OR “interactive agent*”tiab OR “virtual character*”tiab OR “virtual human*”tiab OR “virtual assistant*”tiab OR VRtiab OR “serious game*”tiab OR “serious gaming”tiab OR gamificationtiab

# Trials/ SR/ Meta-analysis

"Meta-Analysis" Publication Type OR "Meta-Analysis as Topic"Mesh OR metaanaly*tiab OR meta-analy*tiab or metanaly*tiab OR "Systematic Review" Publication Type OR systematicsb OR meta-analysisFilter OR systematicreviewFilter OR "Cochrane Database Syst Rev"Journal or prismatiab OR “preferred reporting items”tiab OR prosperotiab OR ((systemati*ti OR umbrellati OR “structured literature”ti) AND (reviewti OR overviewti)) OR “systematic review”tiab OR “umbrella review”tiab OR “structured literature review”tiab OR “systematic qualitative review”tiab OR “systematic quantitative review”tiab OR “systematic search and review”tiab OR “systematized review”tiab OR “systematised review”tiab OR “systemic review”tiab OR “systematic literature review”tiab OR “systematic integrative literature review”tiab OR “systematically review”tiab OR “scoping literature review”tiab OR “scoping review”tiab OR “systematic critical review”tiab OR “systematic integrative review”tiab OR “systematic evidence review”tiab OR “systematic integrative literature review”tiab OR “systematic mixed studies review”tiab OR “systematized literature review”tiab OR “systematic overview”tiab OR “Systematic narrative review”tiab OR “narrative review”tiab OR metasynthes*tiab OR meta-synthes*tiab OR “Randomized Controlled Trial”pt OR “Randomized Controlled Trials as Topic”Mesh OR “Random allocation” Mesh OR “Double-blind method”Mesh OR “Single-blind method”Mesh OR randomtiab OR randomlytiab OR randomisedtiab OR randomizedtiab OR randomisingtiab OR randomizing tiab OR ((singl*tiab OR doubl*tiab OR trebl*tiab OR tripl*tiab) AND (mask*tiab OR blind*tiab OR dumm*tiab)) OR RCTtiab OR "Clinical Trials as Topic"Mesh OR “Clinical Trial”pt OR “clinical trial*”tiab OR “Controlled Clinical Trial”pt OR “controlled trial*”tiab

# Publication type

NOT ("Comment" Publication Type OR "Letter" Publication Type OR "Editorial" Publication Type OR (("Animals"Mesh OR "Models, Animal"Mesh) NOT "Humans"Mesh))

# Timeframe

After 2000

# B. Modified manual of Cochrane risk of bias Tool, version 2.

The modified manual of the Cochrane Risk of Bias tool was adjusted based on the Cochrane Risk of Bias, version 2 (Sterne et al., 2019). Below, we summarized the adaptions made to the modified RoB tool used in the paper.

1. **Imbalance in baseline clinical severity between groups:**

Imbalance in baseline depression severity was examined by the SMD at baseline and its 99% confidence intervals. The SMD > 0.2 (with 99% CI lower limit > 0) is suggestive of baseline imbalance. We focused on baseline depression only, because this is the consistently reported and strong predictor of the outcome. Bonferroni corrections were applied.

1. **Appropriate analysis used for handling the impact of missing data:**

The following analyses was considered LOW RISK.

- MMRM (mixed models for repeated measures, also known as mixed models, growth curve analyses) based on two or more measurements after baseline (e.g., mid-treatment, post-test, follow-ups, etc.)
- Sensitivity analyses corresponding with a range of plausible reasons for missingness to confirm the primary analyses. Such rarely happens in psychotherapy trials.
- The MMRM data (e.g., means) should be reported in the paper

1. **Are outcomes of interest for the meta-analysis in line fully reported in the paper or available through other means? (e.g., contact with authors)**

This was rated depending on the meta-analysis protocol. If meta-analysts aimed to include all available instruments for a given outcome (e.g., depression severity), the article should report all instruments in full (with enough data to be entered in the meta-analysis). If meta-analysts had a pre-specified hierarchy for instrument inclusion (e.g., select HAM-D over BDI), then selective reporting was judged regarding the availability of outcomes based on the pre-specified hierarchy. It will also be rated as high risk when a non-registered outcome was added in the publication of the paper.

Example of details for the depression database:

- All depression outcomes should be reported in full in the paper with enough data to be entered in the meta-analysis. If that was the case, LOW RISK
- If a pre-specified depression measure was not reported in full, HIGH RISK
- If a non-registered or pre-specified outcome was added to the paper, HIGH RISK
- If we obtained the data from IPD or contact through authors, LOW RISK

**Overall score:**

- **Low risk:** if all domains low risk
- **High risk** :if a trial has a high risk in at least one domain or multiple some concerns (e.g., more than **three some concerns)**
- **Some concerns:** if at least one domain has some concerns and max 2 domains with some concerns

# C. Supplemented Tables & Figures

## Table S1. selected characteristics of included studies.

| **Study** | **Intervention** | **Contorl** | **N_ig** | **N_ctr** | **%Female** | **%Male** | **%Other sex** | **Target** | **Recruitment** | **Program name** | **Format** | **N sessions** | **Commerical available** | **Forum** | **Inital human screening** | **Support** | **Country** |
| --- | --- | --- | --- | --- | --- | --- | --- | --- | --- | --- | --- | --- | --- | --- | --- | --- | --- |
| **Addington, 2019** | other psy | wl | 26 | 15 | 0.66 | 0.33 | 0.02 | adul | com | MARIGOLD | internet | 8 | NI | n | y | pure | USA |
| **Addington, 2019** | other psy | other ctr | 26 | 17 | 0.66 | 0.33 | 0.02 | adul | com | MARIGOLD | internet | 8 | NI | n | y | pure | USA |
| **Arean, 2016** | pst | other ctr | nr | nr | 0.79 | 0.21 | nr | adul | com | IPST | mobile | nr | n | n | n | tech | USA |
| **Bedford, 2018** | pst | wl | 12 | 12 | 0.21 | 0.79 | nr | oth | oth | ePST® | computer | 6 | y | n | y | auto | USA |
| **Beevers, 2017** | cbt | wl | 285 | 91 | 0.74 | 0.25 | 0.01 | adul | com | Deprexis | internet | 10 | y | n | y | auto | USA |
| **Berger, 2011** | cbt | wl | 25 | 26 | 0.70 | 0.30 | nr | adul | com | Deprexis | internet | 10 | y | n | y | pure | Europe |
| **Bilich, 2008** | cbt | wl | 17 | 34 | 0.70 | 0.30 | nr | adul | com | The Good Mood Guide: A Self-Help Manual for Depression | book | 8 | n | n | y | human | Australia |
| **Birney, 2016** | cbt | other ctr | nr | nr | 0.77 | 0.23 | nr | oth | com | Moodhacker | mobile | 6 | y | n | y | auto | USA |
| **Blanco, 2023** | cbt | other ctr | 29 | 30 | 0.92 | 0.08 | nr | oth | oth | app-based cognitive–behavioral intervention | mobile | 5 | n | n | y | auto | Europe |
| **Bowman, 1995** | pst | wl | 10 | 10 | 0.63 | 0.37 | nr | adul | com | self-examination booklet | book | nr | NI | n | y | human | USA |
| **Bowman, 1995** | cbt | wl | 10 | 10 | 0.63 | 0.37 | nr | adul | com | Feeling good | book | 20 | y | n | y | human | USA |
| **Christensen, 2004** | cbt | other ctr | nr | nr | 0.71 | 0.29 | nr | adul | com | MoodGym | internet | 5 | n | n | n | tech | Australia |
| **Christensen, 2004** | cbt | other ctr | nr | nr | 0.71 | 0.29 | nr | adul | com | BluePages | internet | 5 | n | n | n | tech | Australia |
| **Clarke, 2019** | cbt | other ctr | 232 | 241 | 0.64 | 0.36 | nr | med | com | myCompass | internet | 3 | n | n | n | auto | Australia |
| **Cooper, 2011** | cbt | cau | 9 | 12 | 0.75 | 0.25 | nr | med | oth | Beating the blues | internet | 8 | y | n | y | pure | UK |
| **Dahne, 2019a** | bat | cau | nr | nr | 0.67 | 0.33 | nr | oth | com | !Aptivate! | mobile | nr | n | n | y | demand | USA |
| **Dahne, 2019a** | cbt | cau | nr | nr | 0.67 | 0.33 | nr | oth | com | iCouch | mobile | nr | n | n | y | tech | USA |
| **Dahne, 2019b** | bat | cau | nr | nr | 0.85 | 0.15 | nr | adul | com | Moodivate | mobile | nr | n | n | y | tech | USA |
| **Dahne, 2019b** | cbt | cau | nr | nr | 0.85 | 0.15 | nr | adul | com | Moodkit | mobile | nr | n | n | y | tech | USA |
| **Dahne, 2023** | bat | other ctr | nr | nr | 0.53 | 0.47 | nr | adul | com | Goal2Quit | mobile | nr | n | n | n | pure | USA |
| **Danaher, 2022** | cbt | cau | 96 | 95 | 1.00 | 0.00 | nr | ppd | oth | MomMoodBooster2 | internet | 6 | y | n | y | tech | USA |
| **de Graaf, 2009** | cbt | cau | 100 | 103 | 0.57 | 0.43 | nr | adul | oth | Colour your life | internet | 8 | n | n | y | pure | Europe |
| **de Graaf, 2009** | cbt | cau | 100 | 103 | 0.57 | 0.43 | nr | adul | oth | Colour your life + cau | internet | 8 | n | n | y | pure | Europe |
| **Deady, 2016** | cbt | other ctr | 30 | 26 | 0.60 | 0.40 | nr | oth | com | DEAL project | internet | 4 | n | n | n | pure | Australia |
| **Ebert, 2018** | other psy | wl | 102 | 102 | 0.80 | 0.20 | nr | adul | com | GET.ON Mood Enhancer | internet | 6 | n | n | y | human | Europe |
| **Farrer, 2011** | cbt | other ctr | 27 | 33 | 0.82 | 0.18 | nr | adul | clin | BluePages + MoodGym (web only) | internet | 5 | n | n | y | demand | Australia |
| **Farrer, 2011** | cbt | other ctr | 18 | 33 | 0.82 | 0.18 | nr | adul | clin | BluePages + MoodGym (web with tracking) | internet | 5 | n | n | y | human | Australia |
| **Farrer, 2011** | cbt | cau | 27 | 27 | 0.82 | 0.18 | nr | adul | clin | BluePages + MoodGym (web only) | internet | 5 | n | n | y | demand | Australia |
| **Farrer, 2011** | cbt | cau | 18 | 27 | 0.82 | 0.18 | nr | adul | clin | BluePages + MoodGym (web with tracking) | internet | 5 | n | n | y | human | Australia |
| **Floyd, 2004** | cbt | wl | 13 | 14 | 0.76 | 0.24 | nr | old | com | Feeling good | book | 20 | y | n | y | human | USA |
| **Fonseca, 2020** | cbt | wl | 98 | 96 | 1.00 | 0.00 | nr | ppd | com | BeaMom | internet | 5 | NI | n | y | tech | Europe |
| **Garnefski, 2011** | cbt | wl | 15 | 17 | 0.85 | 0.15 | nr | med | com | Cognitive-Behavioral Self-help program (CBS) | other | nr | n | n | n | pure | Europe |
| **Gaudiano, 2020** | 3rd | other ctr | 21 | 19 | 0.90 | 0.10 | nr | adul | com | LifeStories | video | 4 | n | n | y | pure | USA |
| **Ghosh, 2021** | cbt | wl | nr | nr | 0.20 | 0.80 | nr | adul | com | plain-text version | internet | 6 | n | n | n | tech | Other |
| **Ghosh, 2021** | cbt | wl | nr | nr | 0.20 | 0.80 | nr | adul | com | TreadWill | internet | 6 | n | y | n | auto | Other |
| **Gilbody, 2015** | cbt | cau | nr | nr | 0.67 | 0.33 | nr | adul | clin | Beating the blues | internet | 8 | y | n | y | human | UK |
| **Gilbody, 2015** | cbt | cau | nr | nr | 0.67 | 0.33 | nr | adul | clin | MoodGym | internet | 5 | n | n | y | human | UK |
| **Gilbody, 2021** | bat | cau | 137 | 141 | 0.55 | 0.43 | 0.02 | old | clin | SHARD self-help booklet | book | nr | n | n | y | human | UK |
| **Gili, 2020** | other psy | cau | nr | nr | 0.78 | 0.22 | nr | adul | clin | iPAPP | internet | 4 | n | n | y | auto | Europe |
| **Glozier, 2013** | cbt | other ctr | 214 | 273 | 0.61 | 0.39 | nr | med | oth | eCouch | internet | 12 | n | n | n | tech | Australia |
| **Gold, 2023** | cbt | wl | nr | nr | 0.77 | 0.23 | nr | med | oth | Amiria | internet | 10 | y | n | n | pure | eu&us |
| **Gräfe, 2020** | cbt | cau | nr | nr | 0.79 | 0.21 | nr | adul | oth | Deprexis | internet | 10 | y | n | n | pure | Europe |
| **Griffiths, 2012** | other psy | other ctr | nr | nr | 0.68 | 0.32 | nr | adul | com | eCouch | internet | 12 | n | n | n | pure | Australia |
| **Griffiths, 2012** | other psy | other ctr | nr | nr | 0.68 | 0.32 | nr | adul | com | WellBeing Board | internet | 12 | n | y | n | pure | Australia |
| **Griffiths, 2012** | other psy | other ctr | nr | nr | 0.68 | 0.32 | nr | adul | com | eCouch + Wellbeing Board | internet | 12 | n | y | n | pure | Australia |
| **Guertler, 2023** | bat | cau | 227 | 229 | 0.63 | 0.37 | nr | adul | oth | ActiLife | other | nr | n | n | y | auto | Europe |
| **Guo, 2020** | other psy | wl | 150 | 150 | 0.77 | 0.23 | nr | med | oth | Run4Love | mobile | 12 | NI | n | y | human | East Asia |
| **Gupta, 2020** | cbt | cau | nr | nr | 0.17 | 0.83 | nr | med | oth | Beating the Blues | internet | 8 | y | n | y | human | USA |
| **Harrer, 2021** | other psy | other ctr | 100 | 100 | 0.85 | 0.15 | nr | stud | com | StudiCare Fernstudierende | internet | 7 | n | n | n | demand | Europe |
| **He, 2022** | cbt | other ctr | 49 | 50 | 0.37 | 0.63 | nr | stud | com | XiaoE | mobile | 7 | NI | n | y | tech | East Asia |
| **He, 2022** | other psy | other ctr | 49 | 50 | 0.37 | 0.63 | nr | stud | com | I Had A Black Dog | book | 7 | n | n | y | pure | East Asia |
| **Hobfoll, 2016** | cbt | wl | 209 | 94 | 0.18 | 0.82 | nr | oth | oth | Vets Prevail | internet | 7 | n | y | y | human | USA |
| **Hur, 2018** | cbt | other ctr | 17 | 17 | 0.88 | 0.12 | nr | adul | com | Todac Todac | mobile | nr | NI | y | y | pure | East Asia |
| **Jamison, 1995** | cbt | wl | 33 | 39 | 0.84 | 0.16 | nr | adul | com | Feeling good | book | 20 | y | n | y | human | USA |
| **Jannati, 2020** | cbt | wl | 39 | 39 | 1.00 | 0.00 | nr | ppd | com | Happy MOM | mobile | 8 | n | n | y | pure | Other |
| **Jelinek, 2020** | bat | other ctr | 29 | 27 | 0.77 | 0.23 | nr | adul | com | iBA | internet | nr | n | n | n | pure | Europe |
| **Jelinek, 2020** | bat | cau | 29 | 32 | 0.77 | 0.23 | nr | adul | com | iBA | internet | nr | n | n | n | pure | Europe |
| **Krämer, 2022** | cbt | other ctr | 150 | 100 | 0.83 | 0.17 | nr | adul | com | Selfapy | internet | 12 | y | n | y | tech | Europe |
| **Lambert, 2018** | bat | wl | 25 | 25 | 0.84 | 0.16 | nr | adul | com | eMotion | internet | 13 | n | n | n | human | UK |
| **Levesque, 2011** | other psy | other ctr | nr | nr | 0.67 | 0.33 | nr | adul | clin | computer-tailored intervention (CTI) | other | nr | n | n | y | auto | USA |
| **Levin, 2011** | cbt | cau | 99 | 91 | 0.77 | 0.23 | nr | adul | oth | Wellness Workshop (WW) | computer | 5 | y | n | y | human | USA |
| **Lin, 2023** | cbt | wl | 37 | 43 | 0.74 | 0.26 | nr | adul | clin | Morning Mood | mobile | 7 | n | n | y | human | East Asia |
| **Lintvedt, 2013** | cbt | wl | 81 | 82 | 0.77 | 0.23 | nr | stud | com | MoodGym + Bluepages | internet | 5 | n | n | n | pure | Europe |
| **Liu, 2009** | cbt | wl | 27 | 25 | 0.73 | 0.27 | nr | adul | com | Mind over mood (chinese version) | book | 10 | y | n | n | demand | East Asia |
| **Lobner, 2018** | cbt | cau | 246 | 296 | 0.68 | 0.32 | nr | adul | clin | MoodGym | internet | 5 | n | n | y | pure | Europe |
| **Lobner, 2018** | cbt | cau | 255 | 296 | 0.68 | 0.32 | nr | adul | clin | MoodGym | internet | 5 | n | n | y | pure | Europe |
| **Lokman, 2017** | cbt | wl | 165 | 164 | 0.76 | 0.24 | nr | adul | com | complaint­directed mini­interventions (CDMIs) | internet | 4 | n | n | n | demand | Europe |
| **Lukas, 2021** | other psy | wl | 40 | 37 | 0.73 | 0.27 | nr | adul | com | mentalis Phoenix (MT-Phoenix) | mobile | 13 | y | n | n | auto | Europe |
| **Mason, 2022** | cbt | wl | nr | nr | 0.83 | 0.17 | nr | yadul | com | text-based CBT | other | nr | n | n | n | demand | USA |
| **Meyer, 2015** | cbt | wl | 78 | 85 | 0.75 | 0.25 | nr | adul | oth | Deprexis | internet | 10 | y | n | y | auto | Europe |
| **Meyer, 2019** | cbt | wl | 100 | 100 | 0.64 | 0.36 | nr | med | com | Emyna | internet | 5 | y | n | y | pure | Europe |
| **Milgrom, 2016** | cbt | cau | 21 | 22 | 1.00 | 0.00 | nr | ppd | com | MumMoodBooster | internet | 6 | n | y | y | human | Australia |
| **Milgrom, 2021** | cbt | cau | 39 | 38 | 1.00 | 0.00 | nr | ppd | com | MumMoodBooster | internet | 6 | n | n | y | human | Australia |
| **Mohr, 2013** | cbt | wl | 35 | 33 | 0.72 | 0.28 | nr | adul | com | moodManager | internet | 18 | n | n | y | pure | USA |
| **Mohr, 2013** | cbt | wl | 34 | 33 | 0.72 | 0.28 | nr | adul | com | moodManager+telecoaching | internet | 18 | n | n | y | human | USA |
| **Moldovan, 2013** | cbt | wl | 21 | 22 | 0.88 | 0.12 | nr | stud | com | Feeling good | book | 20 | y | n | y | human | Europe |
| **Moldovan, 2013** | cbt | cau | 21 | 20 | 0.88 | 0.12 | nr | stud | com | Feeling good | book | 20 | y | n | y | human | Europe |
| **Moldovan, 2013** | cbt | other ctr | 21 | 21 | 0.88 | 0.12 | nr | stud | com | Feeling good | book | 20 | y | n | y | human | Europe |
| **Montero-Marin, 2016** | cbt | cau | 57 | 67 | 0.76 | 0.24 | nr | adul | clin | Smiling is Fun | internet | 10 | n | n | y | auto | Europe |
| **Morgan, 2012** | other psy | other ctr | 276 | 293 | 0.78 | 0.22 | nr | adul | com | Mood Memos | internet | 12 | n | n | n | pure | Australia |
| **Naeem, 2013** | cbt | cau | 94 | 89 | 0.56 | 0.44 | nr | adul | clin | culturally adapted CBT therapy manual | book | 7 | n | n | y | human | Other |
| **Naylor, 2010** | cbt | cau | 15 | 18 | 0.84 | 0.16 | nr | adul | clin | Feeling Good | book | 20 | y | n | y | human | USA |
| **O'Mahen, 2013** | bat | cau | 181 | 162 | 1.00 | 0.00 | nr | adul | com | NetMums | internet | 11 | n | y | n | demand | UK |
| **O'Moore, 2018** | cbt | cau | 44 | 25 | 0.80 | 0.20 | nr | med | oth | Sadness Program | internet | 6 | n | n | y | pure | Australia |
| **Pardini, 2014 (study 1)** | cbt | wl | 13 | 10 | 0.22 | 0.78 | nr | oth | oth | Feeling Good | book | 20 | y | n | y | pure | USA |
| **Pardini, 2014 (study 2)** | cbt | wl | 19 | 23 | 0.00 | 1.00 | nr | oth | oth | Feeling Good | book | 20 | y | n | y | pure | USA |
| **Phillips, 2014** | cbt | other ctr | 164 | 176 | 0.51 | 0.47 | 0.02 | oth | oth | MoodGym | internet | 5 | n | n | y | human | UK |
| **Roepke, 2015** | cbt | wl | 20 | 36 | 0.71 | 0.29 | nr | adul | com | SuperBetter for depression | internet | nr | n | y | n | pure | USA |
| **Roepke, 2015** | 3rd | wl | 18 | 36 | 0.71 | 0.29 | nr | adul | com | general SuperBetter | internet | nr | y | y | n | pure | USA |
| **Rohde, 2014** | cbt | other ctr | 21 | 29 | 0.70 | 0.31 | nr | stud | com | Feeling good | book | 20 | y | n | n | pure | USA |
| **Rosso, 2017** | cbt | other ctr | 37 | 40 | 0.69 | 0.31 | nr | adul | com | Sadness Program | internet | 6 | n | n | y | human | USA |
| **Ruehlman, 2021** | cbt | wl | 28 | 24 | 0.58 | 0.42 | nr | stud | com | TCD-BITS | internet | 4 | n | n | n | pure | USA |
| **Salkovskis, 2006** | other psy | cau | 50 | 46 | 0.80 | 0.20 | nr | adul | clin | CarePartners Programme | book | 6 | n | n | y | pure | UK |
| **Sandoval, 2017** | pst | wl | 25 | 20 | 0.62 | 0.38 | nr | adul | com | imbPST | computer | 6 | NI | n | y | auto | USA |
| **Schmidt, 1983** | cbt | wl | 12 | 10 | 0.84 | 0.16 | nr | adul | com | sel-help therapy book | book | nr | NI | n | y | human | USA |
| **Schure, 2019** | cbt | wl | 181 | 162 | 0.85 | 0.15 | nr | adul | com | Thrive | internet | 3 | y | n | n | auto | USA |
| **Scogin, 1989** | cbt | wl | 21 | 21 | 0.85 | 0.15 | nr | old | com | Feeling good | book | 20 | y | n | y | human | USA |
| **Scogin, 1989** | cbt | wl | 20 | 21 | 0.85 | 0.15 | nr | old | com | Control your depression | book | 7 | y | n | y | human | USA |
| **Selmi, 1990** | cbt | wl | 12 | 12 | 0.64 | 0.36 | nr | adul | com | computer-administered cbt | computer | 6 | n | n | y | demand | USA |
| **Seo, 2022** | cbt | other ctr | 37 | 36 | 1.00 | 0.00 | nr | ppd | com | Happy Mother | mobile | nr | NI | y | y | human | East Asia |
| **Shah, 2018** | cbt | wl | 14 | 12 | 0.84 | 0.16 | nr | old | oth | ACBT | audio | 8 | n | n | n | human | USA |
| **Shah, 2018** | cbt | wl | 14 | 12 | 0.84 | 0.16 | nr | old | oth | CCBT | computer | 11 | n | n | n | human | USA |
| **Shah, 2018** | cbt | wl | 15 | 16 | 0.84 | 0.16 | nr | old | oth | ACBT | audio | 8 | n | n | n | human | USA |
| **Shah, 2018** | cbt | wl | 14 | 16 | 0.84 | 0.16 | nr | old | oth | CCBT | computer | 11 | n | n | n | human | USA |
| **Silverstone, 2017** | cbt | other ctr | 29 | 43 | nr | 1.00 | nr | adul | oth | MoodGym | internet | 5 | n | n | n | pure | Canada |
| **Silverstone, 2017** | cbt | cau | 29 | 48 | nr | 1.00 | nr | adul | oth | MoodGym | internet | 5 | n | n | n | pure | Canada |
| **Smith, 2017** | cbt | wl | 47 | 48 | 0.82 | 0.18 | nr | adul | com | Beating the Blues | book | 12 | n | n | y | pure | Australia |
| **Smith, 2017** | cbt | wl | 33 | 48 | 0.82 | 0.18 | nr | adul | com | Beating the Blues | internet | 6 | n | n | y | human | Australia |
| **Smith, 2017** | cbt | wl | 33 | 48 | 0.82 | 0.18 | nr | adul | com | Beating the Blues | internet | 6 | n | n | y | huamn | Australia |
| **Songprakun, 2012** | cbt | cau | 26 | 28 | 0.73 | 0.27 | nr | adul | clin | The Good Mood Guide: A Self-Help Manual for Depression | book | 8 | n | n | y | human | East Asia |
| **Spek, 2007** | cbt | wl | 102 | 100 | 0.63 | 0.37 | nr | old | com | Coping With Depression (CWD) | internet | 8 | n | n | y | pure | Europe |
| **Stiles-Shields, 2018** | bat | wl | 10 | 10 | nr | 1.00 | nr | adul | com | Boost Me | mobile | 6 | n | n | y | human | USA |
| **Stiles-Shields, 2018** | cbt | wl | 7 | 10 | nr | 1.00 | nr | adul | com | Thought Challenger | mobile | 6 | y | n | y | human | USA |
| **Stuart, 2022** | cbt | cau | 77 | 99 | 0.81 | 0.19 | nr | adul | oth | Thrive | internet | 3 | y | n | n | auto | USA |
| **Sun, 2021** | 3rd | other ctr | 63 | 54 | 1.00 | 0.00 | nr | ppd | oth | Spirits Healing | mobile | 8 | NI | n | y | pure | East Asia |
| **Thitipitchayanant, 2018** | other psy | cau | nr | nr | 1.00 | 0.00 | nr | ppd | oth | SelfEar Program | audio | 4 | NI | n | y | pure | East Asia |
| **Titov, 2010** | cbt | wl | 41 | 40 | 0.74 | 0.26 | nr | adul | com | Sadness Program | internet | 6 | n | n | y | human | Australia |
| **Vázquez, 2023** | cbt | other ctr | 58 | 63 | 0.91 | 0.09 | nr | oth | oth | CBIA | mobile | 5 | n | n | y | pure | Europe |
| **Walker, 2014** | cbt | wl | 21 | 22 | 0.69 | 0.31 | nr | stud | com | CBT homework+with interpersonal element | other | nr | n | y | y | pure | USA |
| **Walker, 2014** | cbt | wl | 22 | 22 | 0.69 | 0.31 | nr | stud | com | CBT homework | other | nr | n | n | y | pure | USA |
| **Walker, 2014** | other psy | wl | 21 | 22 | 0.69 | 0.31 | nr | stud | com | CBT homework | other | nr | n | n | y | pure | USA |
| **Zhao, 2022** | 3rd | wl | 95 | 87 | 0.53 | 0.47 | nr | stud | com | iACT | internet | 6 | n | n | y | auto | East Asia |

N_ig=number of participants in the interventions group, N_ctr=number of participants in the control group. Other sex = diverse sex or missing data. nr=not reported, cbt=cognitive behavior therapy, 3rd=third-wave therapy, pst=problem-solving therapy, bat=behavioral activation therapy, other psy=other kinds of psychotherapy that did not fill into the major category of psychotherapies, wl=wait-list, cau=care as usual, other ctr=other types of controls such as attention control, adul=general adults with no specific demographic characteristics, old= older adults who are above 50 years old, yadul=young adults who has a mean age between 18 to 24 years old, stud=student populations from universities and colleges, ppd= women with postpartum depression, med=people with depression and any general medical disorders, oth=studies aimed at any other specific target group but not included in the other categories, com=community, clin=clinical, oth= other recruitment methods such as screening based on medical records. none=no support, tech=technical support, auto=automatic encouragement generated by the programs, demand=support on demand, human=human encouragement, n=no, y=yes, NI=no information.

## Table S2. Significance test of difference in study characteristics between outliers (n studies=22) and non-outliers (n studies=70).

| **Studies’ characteristics** | | | **Outliers**  **N (%)/M (SD)** | **Non-outliers**  **N (%)/M (SD)** | **Tests** |
| --- | --- | --- | --- | --- | --- |
| Study Design | Control group | WL | 7 (29.2%) | 46 (52.9%) | χ^2^ = 4.831  *p* = .089 |
|  |  | CAU | 10 (41.7%) | 20 (23%) |  |
|  |  | Other | 7 (29.1%) | 21 (24.1%) |  |
|  | Sequence generation | High risk | 1 (4.5%) | 1 (1.4%) | χ^2^ = 3.886  *p* = .143 |
|  |  | Low risk | 20 (91%) | 54 (77.1%) |  |
|  |  | Some concerns | 1 (4.5%)) | 15 (21.5%) |  |
|  | Allocation concealment | High risk | 0 (0%) | 0 (0%) | χ^2^ = 3.115  *p* = .078 |
|  |  | Low risk | 18 (81.8%) | 43 (61.4%) |  |
|  |  | Some concerns | 4 (18.2%) | 27 (38.6%) |  |
|  | Sample size (n>40/arm) | High risk | 6 (27.3%) | 34 (48.6%) | χ^2^ = 3.090  *p* = .079 |
|  |  | Low risk | 16 (72.7%) | 36 (51.4%) |  |
|  |  | Some concerns | 0 (0%) | 0 (0%) |  |
|  | RoB overall rating | High risk | 9 (44.7%) | 36 (51.4%) | χ^2^ = 0.804  *p* = .669 |
|  |  | Low risk | 3 (13.6%)) | 9 (12.9%) |  |
|  |  | Some concerns | 10 (41.7% | 25 (35.7%) |  |
| Analytic method | Intention-to-treat | High risk | 2 (9%) | 24 (47.1%) | χ^2^ = 5.241  *p* = .022 |
|  |  | Low risk | 20 (91%) | 46 (52.9%) |  |
|  |  | Some concerns | 0 (0%) | 0 (0%) |  |
|  | Handle missing data^1^ | High risk | 11 (50%) | 44 (62.9%) | χ^2^ = 1.151  *p* = .283 |
|  |  | Low risk | 11 (50%) | 26 (37.1%) |  |
|  |  | Some concerns | 0 (0%) | 0 (0%) |  |
|  | Selective outcome report | High risk | 13 (55.3%) | 44 (52.9%) | χ^2^ = 0.101  *p* = .751 |
|  |  | Low risk | 9 (44.7%) | 26 (37.1%) |  |
|  |  | Some concerns | 0 (0%) | 0 (0%) |  |
| Participant characteristic | Mean age | | M=38.60  (SD=9.61) | M=39.07  (SD=12.30) | *t* (104) =0.171  *p* = .865 |
|  | Proportion of women | | M=0.65  (SD=0.21) | M=0.73  (SD=0.19) | *t* (106) = 1.729  *p* = .087 |
|  | Target group | Unselected adults | 15 (62.5%) | 49 (56.3%) | χ^2^ = 2.364  *p =* .669 |
|  |  | Student | 2 (8.3%) | 9 (10.3%) |  |
|  |  | Women with PPD | 2 (8.3%) | 6 (6.9%) |  |
|  |  | General medical | 3 (12.6%) | 6 (6.9%) |  |
|  |  | Other | 2 (8.3%) | 17 (19.6%) |  |
|  | Diagnoses method | Cut-off | 15 (62.5%) | 64 (73.6%) | χ^2^ = 1.240  *p =* .538 |
|  |  | Depressive disorder | 7 (29.2%) | 19 (21.8%) |  |
|  |  | Sub-clinical | 2 (8.3%) | 4 (4.6%) |  |
|  | Recruitment method | Community | 11 (45.9%) | 60 (69.1%) | χ^2^ = 4.498  *p= .*105 |
|  |  | Clinical | 5 (20.8%) | 9 (10.3%) |  |
|  |  | Other | 8 (33.3%) | 18 (20.6%) |  |
| Treatment characteristic | Therapy type | CBT | 16 (56.7%) | 63 (72.4%) | χ^2^ = 0.303  *p =* .582 |
|  |  | Other | 8 (33.3%) | 24 (27.6%) |  |
|  | Commercial availability | Yes | 7 (29.2%) | 24 (27.6%) | χ^2^ = 0.023  *p =* .879 |
|  |  | No | 17 (70.8%) | 63 (72.4%) |  |
|  | Delivery Format | Web-based | 14 (58.2%) | 43 (49.4%) | χ^2^ = 2.202  *p = .*699 |
|  |  | Mobile-based | 3 (12.6%) | 16 (18.3%) |  |
|  |  | Computer program | 2 (8.3%) | 3 (3.4%) |  |
|  |  | Bibliotherapy | 3(12.6%) | 17 (19.6%) |  |
|  |  | Other | 2 (8.3%) | 8 (9.3%) |  |
|  | N Sessions | | M=8  (SD=3.79) | M=8.99  (SD=5.10) | *t* (88) = 0.848  *p =* .332 |

CAU=care as usual, WL=waitlist, PPD=postpartum depression, CBT=cognitive behavioral therapy. RoB= Cochrane risk of bias assessment version 2. ^1^Handle missing data refers to whether an appropriate approach was used for handling the impact of missing data. This item was considered low risk in the RoB-2 assessment when the analyses included (1) conducting mixed models for repeated measured based on two or more measurements after baseline, (2) performing multiple imputation based on “Rubin’s rules” or other appropriate methods, or (3) conducting sensitivity analyses to test a range of plausible reasons for missingness

|  | **N** | ***g*** | **95%CI** | ***p*** | ***I^2^*** | **95%CI** | **PI** | **NNT** |
| --- | --- | --- | --- | --- | --- | --- | --- | --- |
| **Correlation coefficient (ρ=0.2)** |  |  |  |  |  |  |  |  |
| Combined (all comparisons) | 111 | 0.55 | 0.46-0.63 | <0.001 | 82.90 | 79.84-85.49 | -0.18-1.27 | 5.43 |
| Outliers removed | 83 | 0.51 | 0.47-0.56 | <0.001 | 25.51 | 1.57-43.63 | 0.28-0.74 | 5.82 |
| Three-Level Model (CHE) | 156 | 0.57 | 0.47-0.66 | <0.001 | 84.40 | - | -0.24-1.37 | 5.23 |
|  |  |  |  |  |  |  |  |  |
| **Correlation coefficient (ρ=0.3)** |  |  |  |  |  |  |  |  |
| Combined (all comparisons) | 111 | 0.54 | 0.46-0.63 | <0.001 | 81.93 | 78.64-84.7 | -0.17-1.26 | 5.47 |
| Outliers removed | 85 | 0.51 | 0.46-0.56 | <0.001 | 28.79 | 6.43-45.81 | 0.26-0.77 | 5.83 |
| Three-Level Model (CHE) | 156 | 0.56 | 0.47-0.65 | <0.001 | 84.30 | - | -0.24-1.37 | 5.25 |
|  |  |  |  |  |  |  |  |  |
| **Correlation coefficient (ρ=0.4)** |  |  |  |  |  |  |  |  |
| Combined (all comparisons) | 111 | 0.54 | 0.46-0.62 | <0.001 | 81.01 | 77.51-83.97 | -0.17-1.25 | 5.51 |
| Outliers removed | 85 | 0.51 | 0.46-0.56 | <0.001 | 26.11 | 2.72-43.88 | 0.27-0.75 | 5.87 |
| Three-Level Model (CHE) | 156 | 0.56 | 0.47-0.65 | <0.001 | 84.30 | - | -0.24-1.37 | 5.27 |
|  |  |  |  |  |  |  |  |  |
| **Correlation coefficient (ρ=0.5)** |  |  |  |  |  |  |  |  |
| Combined (all comparisons) | 111 | 0.54 | 0.46-0.62 | <0.001 | 80.11 | 76.39-83.24 | -0.16-1.24 | 5.55 |
| Outliers removed | 86 | 0.52 | 0.47-0.56 | <0.001 | 26.84 | 3.88-44.32 | 0.26-0.77 | 5.81 |
| Three-Level Model (CHE) | 156 | 0.56 | 0.47-0.65 | <0.001 | 84.20 | - | -0.24-1.36 | 5.28 |
|  |  |  |  |  |  |  |  |  |
| **Correlation coefficient (ρ=0.6) – the primary outcome** | | | | | | | | |
| Combined (all comparisons) | 111 | 0.53 | 0.45-0.61 | <0.001 | 79.17 | 75.22-82.49 | -0.16-1.22 | 5.61 |
| Outliers removed | 87 | 0.52 | 0.47-0.57 | <0.001 | 26.26 | 3.22-43.82 | 0.27-0.76 | 5.81 |
| Three-Level Model (CHE) | 156 | 0.56 | 0.47-0.65 | <0.001 | 84.30 | - | -0.24-1.36 | 5.29 |
|  |  |  |  |  |  |  |  |  |
| **Correlation coefficient (ρ=0.7)** |  |  |  |  |  |  |  |  |
| Combined (all comparisons) | 111 | 0.53 | 0.45-0.61 | <0.001 | 78.09 | 73.87-81.63 | -0.15-1.2 | 5.68 |
| Outliers removed | 87 | 0.51 | 0.46-0.56 | <0.001 | 23.20 | 0-41.61 | 0.27-0.75 | 5.87 |
| Three-Level Model (CHE) | 156 | 0.56 | 0.47-0.65 | <0.001 | 84.30 | - | -0.25-1.36 | 5.30 |
|  |  |  |  |  |  |  |  |  |
| **Correlation coefficient (ρ=0.8)** |  |  |  |  |  |  |  |  |
| Combined (all comparisons) | 111 | 0.52 | 0.44-0.59 | <0.001 | 76.66 | 72.07-80.49 | -0.14-1.17 | 5.80 |
| Outliers removed | 88 | 0.51 | 0.46-0.56 | <0.001 | 21.92 | 0-40.59 | 0.28-0.74 | 5.89 |
| Three-Level Model (CHE) | 156 | 0.56 | 0.47-0.65 | <0.001 | 84.40 | - | -0.25-1.37 | 5.30 |

## Table S3. Sensitivity analyses testing the impact of different correlation coefficients on the pooled effect sizes.

N=numbers of comparisons, CI=confidence interval, PI=prediction interval, Three-Level Model (CHE)=three-level correlated and hierarchical effects (CHE) model.

## Figure S1. Forest plot.


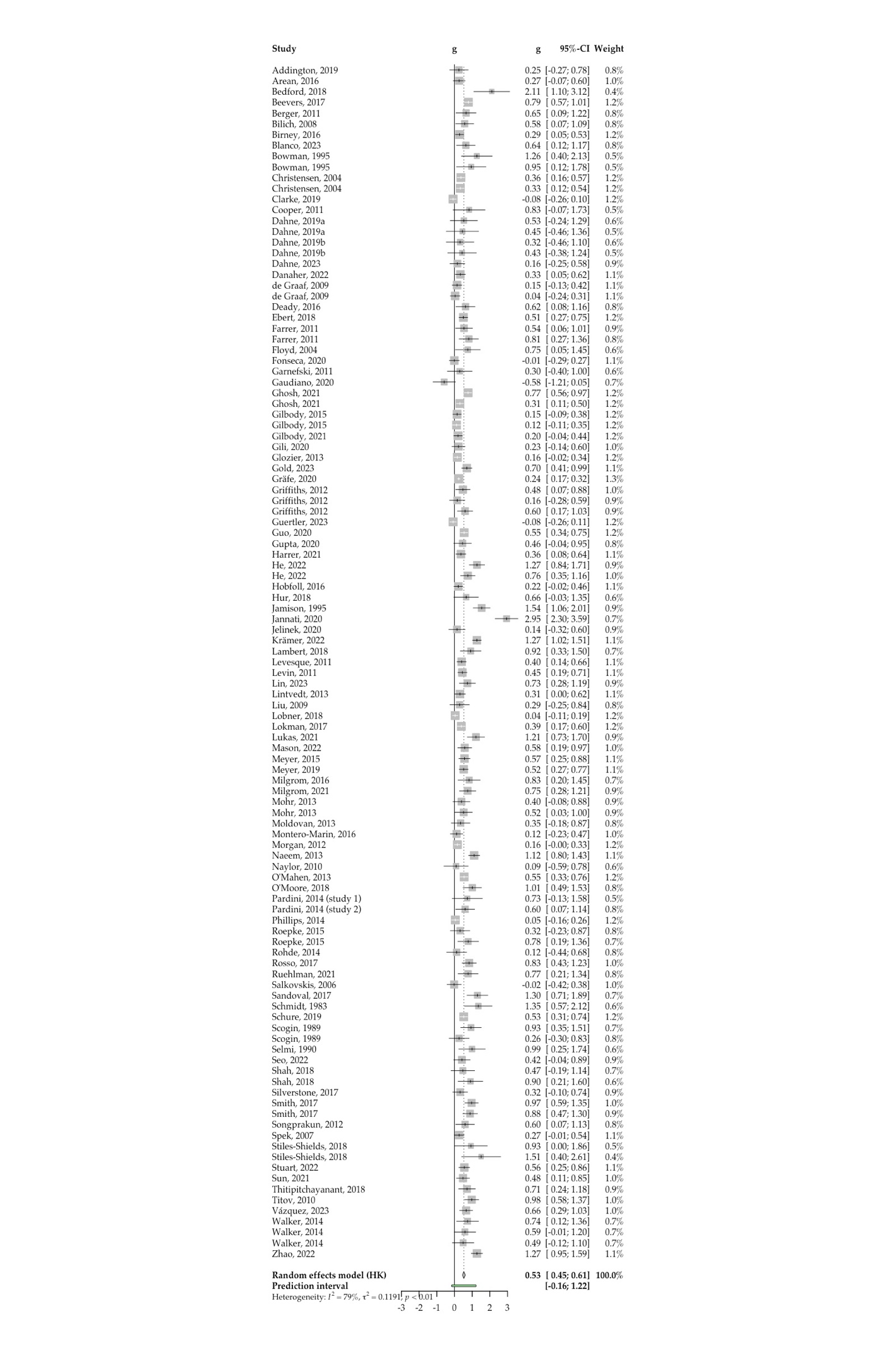


## Figure S2. Funnel plot.

**
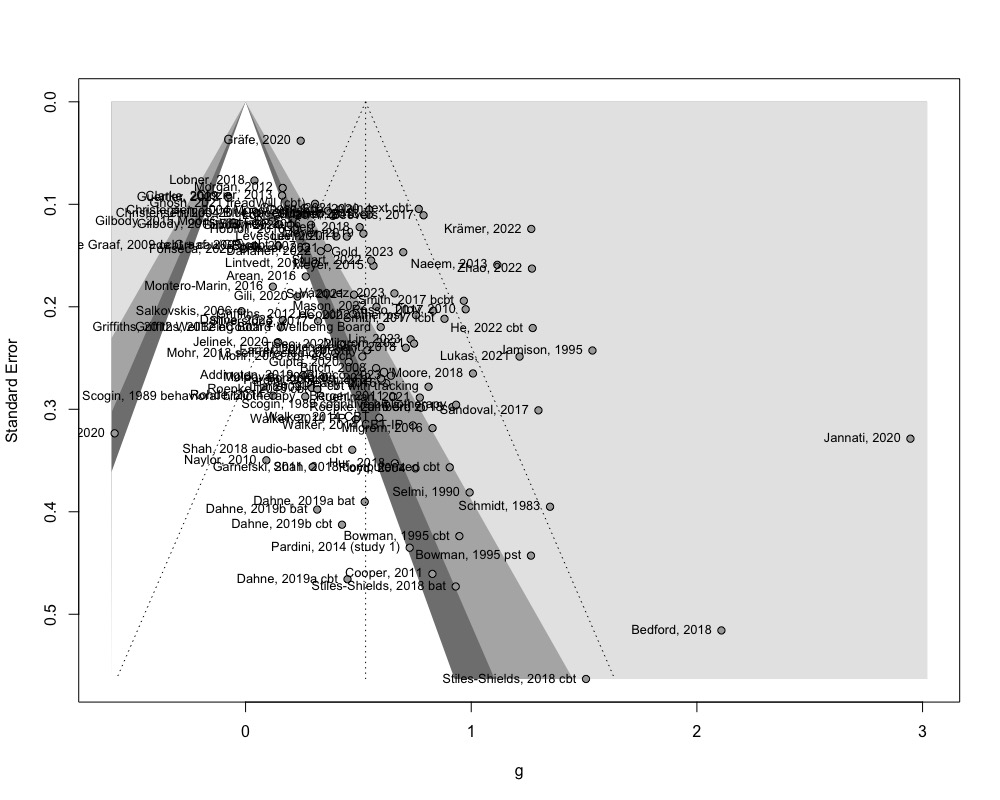
**

## Figure S3. Risk of Bias Summary Plot.


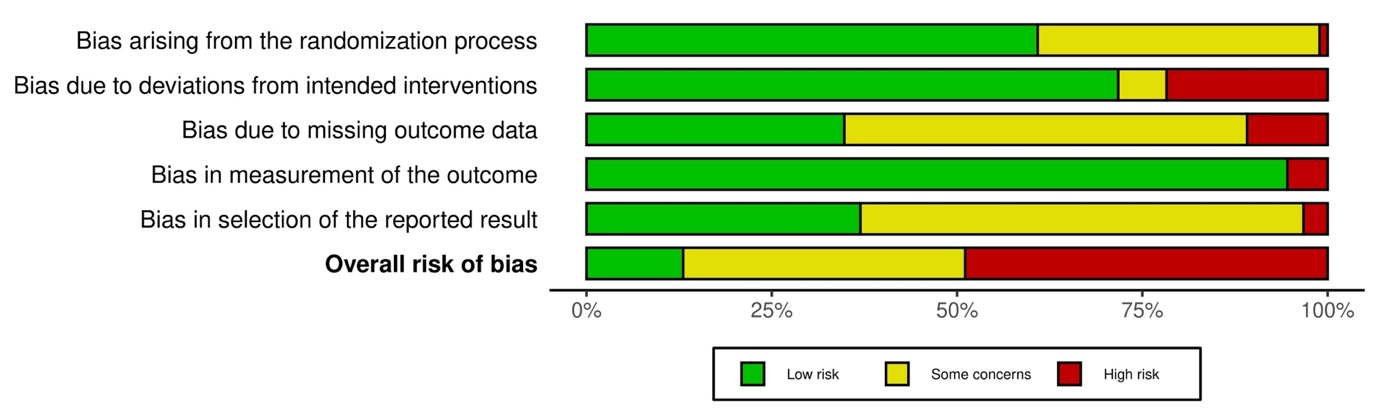


## Figure S4. Risk of Bias traffic light plot.


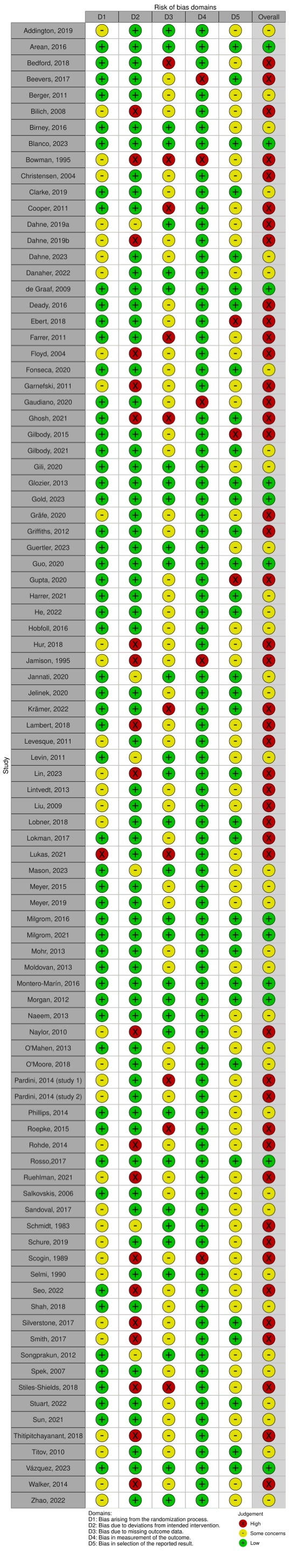


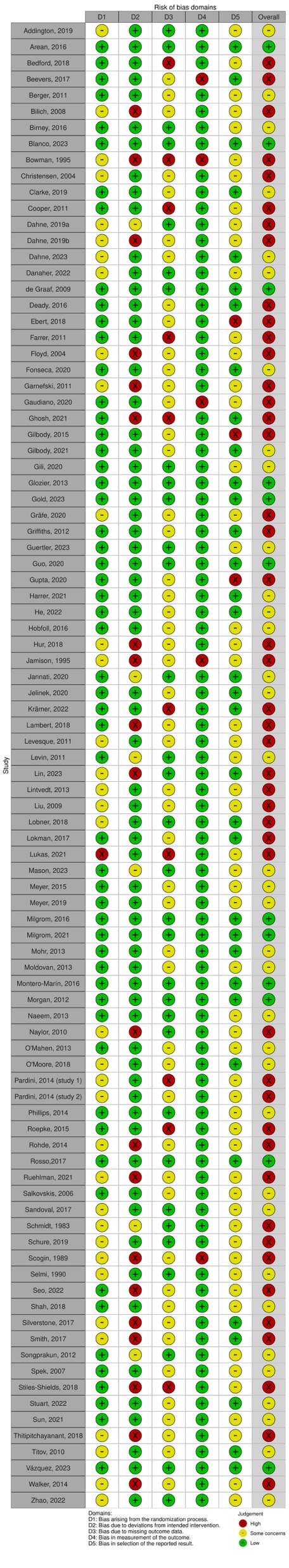


# d. PRISMA CHEKLIST

| **Section and Topic** | **Item #** | **Checklist item** | **Location where item is reported** |
| --- | --- | --- | --- |
| **TITLE** | | |  |
| Title | 1 | Identify the report as a systematic review. | p.1 |
| **ABSTRACT** | | |  |
| Abstract | 2 | See the PRISMA 2020 for Abstracts checklist. | pp.2-3 |
| **INTRODUCTION** | | |  |
| Rationale | 3 | Describe the rationale for the review in the context of existing knowledge. | pp.6-7 |
| Objectives | 4 | Provide an explicit statement of the objective(s) or question(s) the review addresses. | p.7 |
| **METHODS** | | |  |
| Eligibility criteria | 5 | Specify the inclusion and exclusion criteria for the review and how studies were grouped for the syntheses. | pp.7-8 |
| Information sources | 6 | Specify all databases, registers, websites, organisations, reference lists and other sources searched or consulted to identify studies. Specify the date when each source was last searched or consulted. | p.7 |
| Search strategy | 7 | Present the full search strategies for all databases, registers and websites, including any filters and limits used. | suppl pp.2-6 |
| Selection process | 8 | Specify the methods used to decide whether a study met the inclusion criteria of the review, including how many reviewers screened each record and each report retrieved, whether they worked independently, and if applicable, details of automation tools used in the process. | pp.7-8 |
| Data collection process | 9 | Specify the methods used to collect data from reports, including how many reviewers collected data from each report, whether they worked independently, any processes for obtaining or confirming data from study investigators, and if applicable, details of automation tools used in the process. | pp.8-9 |
| Data items | 10a | List and define all outcomes for which data were sought. Specify whether all results that were compatible with each outcome domain in each study were sought (e.g. for all measures, time points, analyses), and if not, the methods used to decide which results to collect. | pp.9-10 |
|  | 10b | List and define all other variables for which data were sought (e.g. participant and intervention characteristics, funding sources). Describe any assumptions made about any missing or unclear information. | pp.9-10 |
| Study risk of bias assessment | 11 | Specify the methods used to assess risk of bias in the included studies, including details of the tool(s) used, how many reviewers assessed each study and whether they worked independently, and if applicable, details of automation tools used in the process. | p.9 & suppl pp.7-8 |
| Effect measures | 12 | Specify for each outcome the effect measure(s) (e.g. risk ratio, mean difference) used in the synthesis or presentation of results. | p.10 |
| Synthesis methods | 13a | Describe the processes used to decide which studies were eligible for each synthesis (e.g. tabulating the study intervention characteristics and comparing against the planned groups for each synthesis (item #5)). | p.10 |
|  | 13b | Describe any methods required to prepare the data for presentation or synthesis, such as handling of missing summary statistics, or data conversions. | pp.10-11 |
|  | 13c | Describe any methods used to tabulate or visually display results of individual studies and syntheses. | pp.10-11 |
|  | 13d | Describe any methods used to synthesize results and provide a rationale for the choice(s). If meta-analysis was performed, describe the model(s), method(s) to identify the presence and extent of statistical heterogeneity, and software package(s) used. | pp.10-11 |
|  | 13e | Describe any methods used to explore possible causes of heterogeneity among study results (e.g. subgroup analysis, meta-regression). | p.11 |
|  | 13f | Describe any sensitivity analyses conducted to assess robustness of the synthesized results. | pp.10-11 |
| Reporting bias assessment | 14 | Describe any methods used to assess risk of bias due to missing results in a synthesis (arising from reporting biases). | p.9 & suppl pp.7-8 |
| Certainty assessment | 15 | Describe any methods used to assess certainty (or confidence) in the body of evidence for an outcome. | - |
| **RESULTS** | | |  |
| Study selection | 16a | Describe the results of the search and selection process, from the number of records identified in the search to the number of studies included in the review, ideally using a flow diagram. | p.12 |
|  | 16b | Cite studies that might appear to meet the inclusion criteria, but which were excluded, and explain why they were excluded. | - |
| Study characteristics | 17 | Cite each included study and present its characteristics. | p.13 & Table S1 |
| Risk of bias in studies | 18 | Present assessments of risk of bias for each included study. | Figure S4 |
| Results of individual studies | 19 | For all outcomes, present, for each study: (a) summary statistics for each group (where appropriate) and (b) an effect estimate and its precision (e.g. confidence/credible interval), ideally using structured tables or plots. | Figure S1 |
| Results of syntheses | 20a | For each synthesis, briefly summarise the characteristics and risk of bias among contributing studies. | pp.13-15 |
|  | 20b | Present results of all statistical syntheses conducted. If meta-analysis was done, present for each the summary estimate and its precision (e.g. confidence/credible interval) and measures of statistical heterogeneity. If comparing groups, describe the direction of the effect. | pp.13-15 |
|  | 20c | Present results of all investigations of possible causes of heterogeneity among study results. | pp.14-15&Table 2&Table S2 |
|  | 20d | Present results of all sensitivity analyses conducted to assess the robustness of the synthesized results. | p.14& &Table 1 & Table S3 |
| Reporting biases | 21 | Present assessments of risk of bias due to missing results (arising from reporting biases) for each synthesis assessed. | p.14 & Table 1 |
| Certainty of evidence | 22 | Present assessments of certainty (or confidence) in the body of evidence for each outcome assessed. | - |
| **DISCUSSION** | | |  |
| Discussion | 23a | Provide a general interpretation of the results in the context of other evidence. | pp.17-18 |
|  | 23b | Discuss any limitations of the evidence included in the review. | pp.18-19 |
|  | 23c | Discuss any limitations of the review processes used. | p.18 |
|  | 23d | Discuss implications of the results for practice, policy, and future research. | p.19 |
| **OTHER INFORMATION** | | |  |
| Registration and protocol | 24a | Provide registration information for the review, including register name and registration number, or state that the review was not registered. | p.2&p.10 |
|  | 24b | Indicate where the review protocol can be accessed, or state that a protocol was not prepared. | p.10 |
|  | 24c | Describe and explain any amendments to information provided at registration or in the protocol. | p.10&p.12 |
| Support | 25 | Describe sources of financial or non-financial support for the review, and the role of the funders or sponsors in the review. | p.3&p.12 |
| Competing interests | 26 | Declare any competing interests of review authors. | p.1&p.20 |
| Availability of data, code and other materials | 27 | Report which of the following are publicly available and where they can be found: template data collection forms-data extracted from included studies-data used for all analyses-analytic code-any other materials used in the review. | p.19 |

*From:*  Page MJ, McKenzie JE, Bossuyt PM, Boutron I, Hoffmann TC, Mulrow CD, et al. The PRISMA 2020 statement: an updated guideline for reporting systematic reviews. BMJ 2021;372:n71. doi: 10.1136/bmj.n71

For more information, visit: <http://www.prisma-statement.org/>

# E. References of included studies

| 1. Addington, E. L., Cheung, E. O., Bassett, S. M., Kwok, I., Schuette, S. A., Shiu, E., Yang, D., Cohn, M. A., Leykin, Y., Saslow, L. R., & Moskowitz, J. T. The MARIGOLD study: Feasibility and enhancement of an online intervention to improve emotion regulation in people with elevated depressive symptoms. J Affect Disord, 257, 352-364. https://www.ncbi.nlm.nih.gov/pmc/articles/PMC6711819/pdf/nihms-1534942.pdf |
| --- |
| 1. Arean, P. A., Hallgren, K. A., Jordan, J. T., Gazzaley, A., Atkins, D. C., Heagerty, P. J., & Anguera, J. A. (2016). The use and effectiveness of mobile apps for depression: results from a fully remote clinical trial. Journal of medical Internet research, 18(12), e330. |
| 1. Bedford LA, Dietch JR, Taylor DJ, Boals A, Zayfert C. Computer-Guided Problem-Solving Treatment for Depression, PTSD, and Insomnia Symptoms in Student Veterans: a Pilot Randomized Controlled Trial. Behavior therapy 2018-49(5): 756‐67. |
| 1. Beevers, C., Pearson, R., Hoffman, J., Foulser, A., Shumake, J., & Meyer, B. (2017). Effectiveness of an internet intervention (Deprexis) for depression in a united states adult sample: a parallel-group pragmatic randomized controlled trial. Journal of Consulting and Clinical Psychology, 85(4), 367-380. |
| 1. Berger T, Hämmerli K, Gubser N, Andersson G, Caspar F. Internet-based treatment of depression: A randomized controlled trial comparing guided with unguided self-help. Cognitive Behaviour Therapy. 2011;40(4):251-66. |
| 1. Bilich, L. L., Deane, F. P., Phipps, A. B., Barisic, M., & Gould, G. (2008). Effectiveness of bibliotherapy self-help for depression with varying levels of telephone helpline support. Clin Psychol Psychother, 15(2), 61-74. doi:10.1002/cpp.562 |
| 1. Birney A, Gunn R, Russell J.K., Ary DV. MoodHacker Mobile Web App With Email for Adults to Self-Manage Mild-to-Moderate Depression: Randomized Controlled Trial. Journal of medical Internet research 2016-4(1): 2291-5222 |
| 1. Blanco V, Otero P, Vázquez FL. A pilot study for a smartphone app for the prevention of depression in non-professional caregivers. Aging Ment Health. 2023 Jan;27(1):166-175. doi: 10.1080/13607863.2022.2056878. Epub 2022 Mar 30. |
| 1. Bowman D, Scogin F, Lyrene B. The efficacy of self-examination therapy and cognitive bibliotherapy in the treatment of mild to moderate depression. Psychotherapy Research. 1995;5(2):131-40. |
| 1. Christensen H, Griffiths KM, Jorm AF. Delivering interventions for depression by using the internet: Randomised controlled trial. BMJ. 2004;328(7434):265. |
| 1. Clarke, J., Sanatkar, S., Baldwin, P. A., Fletcher, S., Gunn, J., Wilhelm, K., Campbell, L., Zwar, N., Harris, M., Lapsley, H., Hadzi-Pavlovic, D., Christensen, H., & Proudfoot, J. (2019). A Web-Based Cognitive Behavior Therapy Intervention to Improve Social and Occupational Functioning in Adults With Type 2 Diabetes (The SpringboarD Trial): Randomized Controlled Trial. J Med Internet Res, 21(5), e12246. |
| 1. Cooper CL, Hind D, Parry GD, Isaac CL, Dimairo M, O'Cathain A, et al. Computerised cognitive behavioural therapy for the treatment of depression in people with multiple sclerosis: External pilot trial. Trials. 2011;12(1):259. |
| 1. Dahne, J., Collado, A., Lejuez, C. W., Risco, C. M., Diaz, V. A., Coles, L., . . . Carpenter, M. J. (2019). Pilot randomized controlled trial of a Spanish-language Behavioral Activation mobile app (¡Aptívate!) for the treatment of depressive symptoms among united states Latinx adults with limited English proficiency. Journal of affective disorders, 250, 210-217. doi:10.1016/j.jad.2019309 |
| 1. Dahne, J., Lejuez, C. W., Diaz, V. A., Player, M. S., Kustanowitz, J., Felton, J. W., & Carpenter, M. J. (2019). Pilot Randomized Trial of a Self-Help Behavioral Activation Mobile App for Utilization in Primary Care. Behav. Ther., 50(4), 817-827. doi:10.1016/j.beth.2018.1203 2. Dahne, J., et al. (2023). "Behavioral Activation–Based Digital Smoking Cessation Intervention for Individuals With Depressive Symptoms: Randomized Clinical Trial." Journal of Medical Internet Research 25. |
| 1. Danaher BG, Seeley JR, Silver RK, Tyler MS, Kim JJ, La Porte LM, Cleveland E, Smith DR, Milgrom J, Gau JM. Trial of a patient-directed eHealth program to ameliorate perinatal depression: the MomMoodBooster2 practical effectiveness study. Am J Obstet Gynecol. 2023 Apr;228(4):453.e1-453.e10. doi: 10.1016/j.ajog.2022927. |
| 1. de Graaf LE, Gerhards SA, Arntz A, Riper H, Metsemakers JF, Evers SM, et al. Clinical effectiveness of online computerised cognitive-behavioural therapy without support for depression in primary care: Randomised trial. The British Journal of Psychiatry. 2009;195(1):73-80. |
| 1. Deady, M., Mills, K. L., Teesson, M., & Kay-Lambkin, F. (2016). An Online Intervention for Co-Occurring Depression and Problematic Alcohol Use in Young People: Primary Outcomes From a Randomized Controlled Trial. J Med Internet Res, 18(3), e71 |
| 1. Ebert, D. D., Buntrock, C., Lehr, D., Smit, F., Riper, H., Baumeister, H., Cuijpers, P., & Berking, M. (2018). Effectiveness of Web- and Mobile-Based Treatment of Subthreshold Depression With Adherence-Focused Guidance: A Single-Blind Randomized Controlled Trial. Behav Ther, 49(1), 71-83. |
| 1. Farrer L, Christensen H, Griffiths KM, Mackinnon A. Internet-based CBT for depression with and without telephone tracking in a national helpline: Randomised controlled trial. PLoS One. 2011;6(11):e28099. |
| 1. Floyd M, Scogin F, McKendree-Smith NL, Floyd DL, Rokke PD. Cognitive therapy for depression: A comparison of individual psychotherapy and bibliotherapy for depressed older adults. Behavior modification. 2004;28(2):297-318. |
| 1. Fonseca A, Alves S, Monteiro F, Gorayeb R, Canavarro MC. Be a Mom, a Web-Based Intervention to Prevent Postpartum Depression: Results From a Pilot Randomized Controlled Trial. Behav Ther. 2020;51(4):616-633. |
| 1. Garnefski N, Kraaij V, Schroevers M. Effects of a cognitive behavioral self-help program on depressed mood for people with acquired chronic physical impairments: A pilot randomized controlled trial. Patient education and counseling. 2011;85(2):304-7. |
| 1. Gaudiano BA, Davis CH, Miller IW, Uebelacker L. Pilot randomized controlled trial of a video self-help intervention for depression based on acceptance and commitment therapy: Feasibility and acceptability. Clinical psychology & psychotherapy. 2020;27(3):396-407. |
| 1. Ghosh, A., et al. (2021). TreadWill: Development and pragmatic randomized controlled trial of an unguided, computerized cognitive behavioral therapy intervention in a lower middle-income country. |
| 1. Gilbody S, Littlewood E, Hewitt C, Brierley G, Tharmanathan P, Araya R, et al. (2015). Computerised cognitive behaviour therapy (cCBT) as treatment for depression in primary care (REEACT trial): Large scale pragmatic randomised controlled trial. |
| 1. Gilbody, S., et al. (2021). "Can We Prevent Depression in At-Risk Older Adults Using Self-Help? The UK SHARD Trial of Behavioral Activation." American Journal of Geriatric Psychiatry. |
| 1. Gili M, Castro A, García-Palacios A, et al. Efficacy of Three Low-Intensity, Internet-Based Psychological Interventions for the Treatment of Depression in Primary Care: Randomized Controlled Trial. J Med Internet Res. 2020;22(6):e15845. |
| 1. Glozier N, Christensen H, Naismith S, Cockayne N, Donkin L, Neal B, et al. Internet-delivered cognitive behavioural therapy for adults with mild to moderate depression and high cardiovascular disease risks: A randomised attention-controlled trial. PLoS One. 2013;8(3):e59139. 2. Gold, S. M., et al. (2023). "Internet-delivered cognitive behavioural therapy programme to reduce depressive symptoms in patients with multiple sclerosis: a multicentre, randomised, controlled, phase 3 trial." The Lancet Digital Health 5(10): e668-e678. |
| 1. Gräfe V, Moritz S, Greiner W. Health economic evaluation of an internet intervention for depression (deprexis), a randomized controlled trial. Health Economics Review. 2020;10(1). |
| 1. Griffiths KM, Mackinnon AJ, Crisp DA, Christensen H, Bennett K, Farrer L. The effectiveness of an online support group for members of the community with depression: A randomised controlled trial. PloS One. 2012;7(12):e53244. 2. Guertler D, Krause K, Moehring A, Bischof G, Batra A, Freyer-Adam J, et al. E-Health intervention for subthreshold depression: Reach and two-year effects of a randomized controlled trial. Journal of Affective Disorders. 2023;339:33-42. |
| 1. Guo Y, Hong YA, Cai W, et al. Effect of a WeChat-Based Intervention (Run4Love) on Depressive Symptoms Among People Living With HIV in China: a Randomized Controlled Trial. Journal of medical Internet research. 2020;22(2):e16715. |
| 1. Gupta SK, Slaven JE, Liu Z, Polanka BM, Freiberg MS, Stewart JC. Effects of internet cognitive-behavioral therapy on depressive symptoms and surrogates of cardiovascular risk in human immunodeficiency virus: A pilot, randomized, controlled trial. Open Forum Infectious Diseases. 2020;7(7). |
| 1. Harrer, M., et al. (2021). "Effect of an internet- and app-based stress intervention compared to online psychoeducation in university students with depressive symptoms: Results of a randomized controlled trial." Internet Interventions 24. |
| 1. He, Y., Yang, L., Zhu, X., Wu, B., Zhang, S., Qian, C., & Tian, T. (2022). Mental Health Chatbot for Young Adults With Depressive Symptoms During the COVID-19 Pandemic: Single-Blind, Three-Arm Randomized Controlled Trial. J Med Internet Res, 24(11), e40719. doi:10.2196/40719 |
| 1. Hobfoll SE, Blais RK, Stevens NR, Walt L, Gengler R. Vets prevail online intervention reduces PTSD and depression in veterans with mild-to-moderate symptoms. Journal of Consulting and Clinical Psychology. 2016;84(1):31-42. |
| 1. Hur, J. W., Kim, B., Park, D., & Choi, S. W. A Scenario-Based Cognitive Behavioral Therapy Mobile App to Reduce Dysfunctional Beliefs in Individuals with Depression: A Randomized Controlled Trial. Telemed J E Health, 24(9), 710-716. |
| 1. Jamison C, Scogin F. The outcome of cognitive bibliotherapy with depressed adults. Journal of Consulting and Clinical Psychology. 1995;63(4):644-50. |
| 1. Jannati N, Mazhari S, Ahmadian L, Mirzaee M. Effectiveness of an app-based cognitive behavioral therapy program for postpartum depression in primary care: A randomized controlled trial. Int J Med Inform. 2020;141:104145. |
| 1. Jelinek L, Arlt S, Moritz S, Schröder J, Westermann S, Cludius B. Brief Web-Based Intervention for Depression: Randomized Controlled Trial on Behavioral Activation. J Med Internet Res. 2020;22(3):e15312. |
| 1. Krämer, R., Köhne-Voll, , L., Schumacher, A., & Köhler, S. Efficacy of an online intervention for treatment of depressive disorders: a three-arm randomized controlled trial comparing guided and unguided self-help with waitlist control. JMIR Form Res. |
| 1. Lambert, J. D., Greaves, C. J., Farr, P., Price, L., Haase, A. M., & Taylor, A. H. Web-Based Intervention Using Behavioral Activation and Physical Activity for Adults With Depression (The eMotion Study): Pilot Randomized Controlled Trial. J Med Internet Res, 20(7), e10112. |
| 1. Levesque, D. A., Van Marter, D. F., Schneider, R. J., Bauer, M. R., Goldberg, D. N., Prochaska, J. O., & Prochaska, J. M. (2011). Randomized trial of a computer-tailored intervention for patients with depression. American Journal of Health Promotion, 26(2), 77-89. |
| 1. Levin W, Campbell DR, McGovern KB, Gau JM, Kosty DB, Seeley JR, et al. A computer-assisted depression intervention in primary care. Psychological Medicine. 2011;41(7):1373-83. |
| 1. Lin, Z., et al. (2023). "The Effect of Internet-Based Cognitive Behavioral Therapy on Major Depressive Disorder: Randomized Controlled Trial." Journal of Medical Internet Research 25(1). 2. Lintvedt OK, Griffiths KM, Sorensen K, ÿstvik AR, Wang CE, Eisemann M, et al. Evaluating the effectiveness and efficacy of unguided internet-based self-help intervention for the prevention of depression: A randomized controlled trial. Clinical Psychology and Psychotherapy. 2013;20(1):10-27. 3. Liu ET-H, Chen W-L, Li Y-H, Wang CH, Mok TJ, Huang HS. Exploring the efficacy of cognitive bibliotherapy and a potential mechanism of change in the treatment of depressive symptoms among the Chinese: A randomized controlled trial. Cognitive Therapy and Research. 2009;33(5):449-61. |
|  |
| 1. Lobner M, Pabst A, Stein J, et al. Computerized cognitive behavior therapy for patients with mild to moderately severe depression in primary care: A pragmatic cluster randomized controlled trial (@ktiv). J Affect Disord 2018-238: 317-26. |
| 1. Lokman, S., Leone, S., Sommers-Spijkerman, M., Poel, A., Smit, F., & Boon, B. (2017). Complaint-Directed Mini-Interventions for Depressive Complaints: a Randomized Controlled Trial of Unguided Web-Based Self-Help Interventions. Journal of medical Internet research, 19(1), e4. |
| 1. Lukas, C. A., et al. (2021). "A gamified smartphone-based intervention for depression: Randomized controlled pilot trial." JMIR Mental Health 8(7). |
| 1. Mason, M. J., Coatsworth, J. D., Zaharakis, N., Russell, M., Wallis, D., Brown, A., & Hale, C. (2022). Treating Young Adult Depression With Text-Delivered Cognitive Behavioral Therapy: A Pilot Randomized Clinical Trial. Behavior Therapy. doi:10.1016/j.beth.2022905 |
| 1. Meyer B, Bierbrodt J, Schroder J, Berger T, Beevers CG, Weiss M, et al. Effects of an Internet intervention (Deprexis) on severe depression symptoms: Randomized controlled trial. Internet Interventions. 2015;2(1):48-59. |
| 1. Meyer, B., Weiss, M., Holtkamp, M., Arnold, S., Brückner, K., Schröder, J., . . . Nestoriuc, Y. (2019). Effects of an epilepsy-specific Internet intervention (Emyna) on depression: Results of the ENCODE randomized controlled trial. Epilepsia, 60(4), 656-668. doi:10.1111/epi.14673 |
| 1. Milgrom J, Danaher BG, Gemmill AW, Holt C, Holt CJ, Seeley JR, et al. Internet cognitive behavioral therapy for women with postnatal depression: A randomized controlled trial of MumMoodBooster. Journal of Medical Internet Research. 2016;18(3):e54. |
| 1. Milgrom, J., et al. (2021). "Internet and Face-to-face Cognitive Behavioral Therapy for Postnatal Depression Compared With Treatment as Usual: Randomized Controlled Trial of MumMoodBooster." J Med Internet Res 23(12): e17185. |
| 1. Mohr DC, Duffecy J, Ho J, Kwasny M, Cai X, Burns MN, et al. A randomized controlled trial evaluating a manualized TeleCoaching protocol for improving adherence to a web-based intervention for the treatment of depression. PLoS One. 2013;8(8):e70086. |
| 1. Moldovan R, Cobeanu O, David D. Cognitive bibliotherapy for mild depressive symptomatology: Randomized clinical trial of efficacy and mechanisms of change. Clinical Psychology and Psychotherapy. 2013;20(6):482-93. |
| 1. Montero-Marín, J., Araya, R., Pérez-Yus, M. C., Mayoral, F., Gili, M., Botella, C., . . . López-Del-Hoyo, Y. (2016). An internet-based intervention for depression in primary Care in Spain: a randomized controlled trial. Journal of medical Internet research, 18(8), e231. |
| 1. Morgan, A. J., Jorm, A. F., & Mackinnon, A. J. (2012). Email-based promotion of self-help for subthreshold depression: Mood Memos randomised controlled trial Multicenter Study-Randomized Controlled Trial-Research Support, Non-U.S. Gov't. British Journal of Psychiatry, 200(5), 412-418. doi:10.1192/bjp.bp.111.101394. |
| 1. Naeem F, Sarhandi I, Gul M, Khalid M, Aslam M, Anbrin A. A multicentre randomised controlled trial of a carer supervised culturally adapted cbt (cacbt) based self-help for depression in pakistan. Journal of Affective Disorders. 2013;156:224-7. |
| 1. Naylor EV, Antonuccio DO, Litt M, Johnson GE, Spogen DR, Williams R, et al. Bibliotherapy as a treatment for depression in primary care. Journal of clinical psychology in medical settings. 2010;17(3):258-71. |
| 1. O'Mahen HA, Woodford J, McGinley J, Warren FC, Richards DA, Lynch TR, et al. Internet-based behavioral activation--treatment for postnatal depression (Netmums): A randomized controlled trial. Journal of Affective Disorders. 2013;150(3):814-22. |
| 1. O'Moore K A, Newby JM, Andrews G, et al. Internet Cognitive-Behavioral Therapy for Depression in Older Adults With Knee Osteoarthritis: A Randomized Controlled Trial. Arthritis Care Res (Hoboken) 2018-70(1): 61-70. |
| 1. Pardini J, Scogin F, Schriver J, Domino M, Wilson D, LaRocca M. Efficacy and process of cognitive bibliotherapy for the treatment of depression in jail and prison inmates. Psychological services 2014-11(2): 141-52. (study1). |
| 1. Pardini J, Scogin F, Schriver J, Domino M, Wilson D, LaRocca M. Efficacy and process of cognitive bibliotherapy for the treatment of depression in jail and prison inmates. Psychological services 2014-11(2): 141-52. (study2). |
| 1. Phillips R, Schneider J, Molosankwe I, Leese M, Foroushani P, Grime P. Randomized controlled trial of computerized cognitive behavioural therapy for depressive symptoms: Effectiveness and costs of a workplace intervention. Psychological Medicine. 2014;44(4):741-52. |
| 1. Roepke AM, Jaffee SR, Riffle OM, McGonigal J, Broome R, Maxwell B. Randomized controlled trial of SuperBetter, a smartphone-based/internet-based self-help tool to reduce depressive symptoms. Games for Health 2015-4(3): 235-46. |
| 1. Rosso, I. M., Killgore, W. D., Olson, E. A., Webb, C. A., Fukunaga, R., Auerbach, R. P., Gogel, H., Buchholz, J. L., & Rauch, S. L. Internet-based cognitive behavior therapy for major depressive disorder: A randomized controlled trial. Depress Anxiety, 34(3), 236-245. https://www.ncbi.nlm.nih.gov/pmc/articles/PMC5540163/pdf/nihms884093.pdf 2. Rohde, P., Stice, E., Shaw, H., & Gau, J. M. (2014). Cognitive-behavioral group depression prevention compared to bibliotherapy and brochure control: Nonsignificant effects in pilot effectiveness trial with college students. Behaviour Research and Therapy, 55(1), 48-53. |
| 1. Ruehlman, L. and P. Karoly (2021). "A pilot test of Internet-delivered brief interactive training sessions for depression: Evaluating dropout, uptake, adherence, and outcome." Journal of American college health : J of ACH: 1-9. |
| 1. Salkovskis P, Rimes K, Stephenson D, Sacks G, Scott J. A randomized controlled trial of the use of self-help materials in addition to standard general practice treatment of depression compared to standard treatment alone. Psychological Medicine. 2006;36(03):325-33. |
| 1. Sandoval, L., Buckey, J., Ainslie, R., Tombari, M., Stone, W., & Hegel, M. (2017). Randomized Controlled Trial of a Computerized Interactive Media-Based Problem Solving Treatment for Depression. Behavior Therapy, 48(3), 413-425. |
| 1. Schmidt MM, Miller WR. Amount of therapist contact and outcome in a multidimensional depression treatment program. Acta Psychiatrica Scandinavica. 1983;67(5):319-32. |
| 1. Schure, M. B., Lindow, J. C., Greist, J. H., Nakonezny, P. A., Bailey, S. J., Bryan, W. L., & Byerly, M. J. (2019). Use of a Fully Automated Internet-Based Cognitive Behavior Therapy Intervention in a Community Population of Adults With Depression Symptoms: Randomized Controlled Trial. Journal of medical Internet research, 21(11), e14754. doi:10.2196/14754 |
| 1. Scogin F, Jamison C, Gochneaur K. Comparative efficacy of cognitive and behavioral bibliotherapy for mildly and moderately depressed older adults. Journal of Consulting and Clinical Psychology. 1989;57(3):403-7. |
| 1. Selmi PM, Klein MH, Greist JH, Sorrell SP, Erdman HP. Computer-administered cognitive-behavioral therapy for depression. American Journal of Psychiatry. 1990;147(1):51-6. 2. Seo, J. M., Kim, S. J., Na, H., Kim, J. H., & Lee, H. (2022). Effectiveness of a Mobile Application for Postpartum Depression Self-Management: Evidence from a Randomised Controlled Trial in South Korea. Healthcare (Basel), 10(11). https://doi.org/doi:10.3390/healthcare10112185 3. Shah, A., Morthland, M., Scogin, F., Presnell, A., DiNapoli, E. A., DeCoster, J., & Yang, X. (2018). Audio and Computer Cognitive Behavioral Therapy for Depressive Symptoms in Older Adults: a Pilot Randomized Controlled Trial. Behavior Therapy, 49(6), 904‐916. |
| 1. Silverstone, P. H., Rittenbach, K., Suen, V. Y. M., Moretzsohn, A., Cribben, I., Bercov, M., . . . Trew, M. (2017). Depression outcomes in adults attending family practice were not improved by screening, stepped-care, or online CBT during a 12-week study when compared to controls in a randomized trial. |
| 1. Smith, J., Newby, J. M., Burston, N., Murphy, M. J., Michael, S., Mackenzie, A., . . . Andrews, G. (2017). Help from home for depression: A randomised controlled trial comparing internet-delivered cognitive behaviour therapy with bibliotherapy for depression. Internet Interventions, 9, 25-37. |
| 1. Songprakun W, McCann TV. Evaluation of a cognitive behavioural self-help manual for reducing depression: A randomized controlled trial. Journal of psychiatric and mental health nursing. 2012;19(7):647-53. |
| 1. Spek V, Nyklicek I, Smits N, Cuijpers P, Riper H, Keyzer J, et al. Internet-based cognitive behavioural therapy for subthreshold depression in people over 50 years old: A randomized controlled clinical trial. Psychological Medicine. 2007;37(12):1797-806. |
| 1. Stiles-Shields, C., Montague, E., Kwasny, M. J., & Mohr, D. C. (2018). Behavioral and cognitive intervention strategies delivered via coached apps for depression: Pilot trial. Psychol Serv, 16(2), 233-238. |
| 1. StuartRoderick, HeidiFischer, SLeitzke, A., DavidaBecker, NeetaSaheba, & JColeman, K. (2022). The Effectiveness of Internet-Based Cognitive Behavioral Therapy for the Treatment of Depression in a Large Real-World Primary Care Practice: A Randomized Trial. The Permanente Journal, 26(3), 53-60. https://doi.org/doi:10.7812/TPP/21.183 |
| 1. Sun, Y., et al. (2021). "Effectiveness of Smartphone-Based Mindfulness Training on Maternal Perinatal Depression: Randomized Controlled Trial." J Med Internet Res 23(1): e23410. |
| 1. Thitipitchayanant K, Somrongthong R, Kumar R, Kanchanakharn N. Effectiveness of self-empowerment-affirmation-relaxation(Self-EAR) program for postpartum blues mothers: A randomize controlled trial. Pakistan Journal of Medical Sciences 2018-34(6): 1488-93. |
| 1. Titov, N., Andrews, G., Davies, M., McIntyre, K., Robinson, E., & Solley, K. Internet treatment for depression: a randomized controlled trial comparing clinician vs. technician assistance. PLoS One, 5(6), e10939. https://www.ncbi.nlm.nih.gov/pmc/articles/PMC2882336/pdf/pone010939.pdf 2. Vázquez, F. L., et al. (2023). "Efficacy of a Cognitive Behavioral Intervention for the Prevention of Depression in Nonprofessional Caregivers Administered through a Smartphone App: A Randomized Controlled Trial." Journal of Clinical Medicine 12(18). |
| 1. Walker JV, Lampropoulos GK. A comparison of self-help (homework) activities for mood enhancement: Results from a brief randomized controlled trial. Journal of Psychotherapy Integration. 2014;24(1):46-64. |
| 1. Zhao, C., Wampold, B. E., Ren, Z., Zhang, L., & Jiang, G. (2022). The efficacy and optimal matching of an internet‐based acceptance and commitment therapy intervention for depressive symptoms among university students: A randomized controlled trial in China. Journal of Clinical Psychology, 78(7), 1354-1375. doi:10.1002/jclp.23329. |
